# Genome-wide association and Mendelian randomization study of blood copper levels and 213 deep phenotypes in humans

**DOI:** 10.1038/s42003-022-03351-7

**Published:** 2022-05-02

**Authors:** Wenjun Yang, Longman Li, Xiuming Feng, Hong Cheng, Xiaoting Ge, Yu Bao, Lulu Huang, Fei Wang, Chaoqun Liu, Xing Chen, Zengnan Mo, Xiaobo Yang

**Affiliations:** 1 Center for Genomic and Personalized Medicine, Guangxi key Laboratory for Genomic and Personalized Medicine, Guangxi Collaborative Innovation Center for Genomic and Personalized Medicine, Guangxi Medical University, Nanning, 530021 Guangxi China; 2 Collaborative Innovation Centre of Regenerative Medicine and Medical BioResource Development and Application, Guangxi Medical University, Nanning, Guangxi 530021 China; 3 Department of Occupational Health and Environmental Health, School of Public Health, Guangxi Medical University, Nanning, Guangxi China; 4 Department of radiotherapy, First Affiliated Hospital of Guangxi Medical University, Nanning, China; 5 Guangxi key Laboratory for Thyroid Tumor Precision Prevention and Treatment, Liuzhou, Guangxi China; 6 Department of Nutrition and Food Hygiene, School of Public Health, Guangxi Medical University, Nanning, Guangxi China; 7 School of Public Health, Guangxi Medical University, Nanning, Guangxi China; 8 Department of Urology, Institute of Urology and Nephrology, First Affiliated Hospital of Guangxi Medical University, Nanning, Guangxi China; 9 Department of Public Health, School of Medicine, Guangxi University of Science and Technology, Liuzhou, Guangxi China

**Keywords:** Population genetics, Computational biology and bioinformatics, Risk factors

## Abstract

Metal elements are present in the human body, and their levels in the blood have important impacts on health. In this study, 2488 Chinese individuals were included in a genome-wide association study of 21 serum metal levels, with approximately 179,000 East Asian individuals in a bidirectional two-sample Mendelian randomization (MR) analysis, and 628,000 Europeans in a two-sample MR analysis. We identified two single nucleotide polymorphisms (SNPs) rs35691438 and rs671 that were significantly associated with serum copper levels (SCLs). The bidirectional two-sample MR analysis in the East Asian population showed that gamma-glutamyl transpeptidase levels have a causal effect on SCLs. SCLs have causal effects on six outcomes, namely risks of esophageal varix, glaucoma, sleep apnea syndrome, and systemic lupus erythematosus, white blood cell count, and usage of drugs affecting bone structure and mineralization. The two-sample MR analyses in the European population showed causal effects of erythrocyte copper levels on risks of carpal tunnel syndrome and compression fracture. Our results provide original insights into the causal relationship between blood metal levels and multiple human phenotypes.

## Abstract (web summary)

A genome-wide association study in a Chinese population identifies SNPs associated with serum copper levels. Mendelian randomization analysis reveals causal effects on multiple human phenotypes in East Asian and European populations.

## Introduction

Metal elements are present in the human body and can be classified as essential metal elements and toxic metal elements. Essential metal elements, such as calcium, copper, iron, manganese, magnesium, selenium, and zinc, are obtained mainly through the daily diet. They play critical roles in normal physiological activities, including oxidation–reduction reactions, catalysis, metabolic regulation, and intercellular signal transduction^1,2^. Toxic metal elements, such as arsenic, cadmium, and lead, usually come from environmental exposure^3,4^. The accumulation of toxic metal elements in the human body can lead to many adverse health effects and even endanger life. In addition to the environmental factors that affect metal levels in the human body, many genetic variants that are significantly related to blood metal levels (BMLs) have been discovered. Numerous genome-wide association studies (GWASs) of European populations found a large number of single nucleotide polymorphisms (SNPs) that were significantly associated with blood levels of calcium, copper, selenium, zinc, iron, magnesium, manganese, cadmium, and mercury^5–9^. However, as far as we know, only a few GWASs on multiple BMLs in East Asian populations have been reported.

Many epidemiological studies have shown that BMLs are closely related to human diseases^10–15^. However, traditional epidemiological studies are often affected by confounding factors, and therefore determining causality can be difficult. Mendelian randomization (MR) studies can avoid the bias caused by the unavoidable confounding factors in traditional epidemiological studies, thereby allowing more robust and reliable causal inferences^16^. The application of MR has helped to uncover the causal relationships between BMLs and human disease phenotypes. Genetically higher serum calcium levels were shown to be associated with a higher risk of coronary artery disease and myocardial infarction^17^. Another study of UK Biobank cohorts found that genetically increased blood levels of both copper and iron were associated with reduced risk of lipid metabolism disorders^18^. A two-sample MR study based on Europeans showed that bone mineral density of certain skeletal locations was influenced by serum calcium and selenium levels^19^. Current GWAS-MR studies of BMLs and their causal effects on human diseases focused mainly on European populations. Considering the genetic differences between European and East Asian populations, a systematic GWAS-MR of BMLs in East Asian population is necessary to provide more reliable data for the diagnosis, treatment, and basic scientific research of clinical diseases.

In this study, we conducted a comprehensive GWAS of multiple serum metal levels in the Chinese population and then performed a cross-ethnic MR study to fully understand the causal effects of BMLs on multiple deep phenotypes in humans. For this, we conducted bidirectional two-sample MR analyses using our GWAS summary statistics of serum metal levels and the GWAS summary statistics from BioBank Japan^20^. In addition, to comprehensively study the causal effects of BMLs on multiple deep phenotypes in different populations, we used the GWAS summary statistics of erythrocytes metal levels from the study of Evans et al.^6^ and the GWAS meta-analysis summary statistics from UK Biobank and FinnGen to conduct two-sample MR analyses.

## Results

### Characteristics of subjects

We totally included 2488 subjects in our present study. After the non-parametric test on the concentration of each metal between the two cohorts, only the concentrations of copper and manganese showed no statistical difference (*P*_copper_ = 0.08 and *P*_manganese_ = 0.96). The basic demographic characteristics and the results of the comparison of serum levels for the same metal in two cohorts of the subjects finally included in the GWAS analysis are shown in Table 1.

**Table 1:** Characteristics of the study populations from FAMHES (*N* = 1800) and MEWHC (*N* = 688).

Characteristics	FAMHES (*n* (%) or median ± IQR)	MEWHC (*n* (%) or median ± IQR)	*P*-value
Age, years	36 ± 16	44 ± 9	<0.001
BMI, kg/m^2^	23.04 ± 4.74	23.24 ± 4.14	0.02
*Sex*			
Male	1800 (100)	468 (68)	
Female	-	220 (32)	
*Smoke status*			0.13
Yes	906 (50)	323 (47)	
No	894 (50)	365 (53)	
*Alcoholic drinking*			<0.001
Yes	1494 (83)	277 (40)	
No	306 (17)	461 (67)	
*Ethnicity*			
Han	1800 (100)	320 (47)	
Other	-	368 (53)	
*Serum/Plasma metals measure*			
Aluminum, µg/L	21.15 ± 18.25 [1775]	18.16 ± 22.34 [678]	<0.001
Arsenic, µg/L	6.84 ± 8.13 [1773]	0.85 ± 0.34 [676]	<0.001
Barium, µg/L	31.70 ± 15.65 [1792]	2.79 ± 13.62 [687]	<0.001
Calcium, mg/L	110.94 ± 9.82 [1740]	102.01 ± 7.53 [688]	<0.001
Cadmium, µg/L	0.07 ± 0.05 [1775]	0.17 ± 0.25 [656]	<0.001
Cobalt, µg/L	0.12 ± 0.05 [1758]	0.12 ± 0.04 [639]	0.03
Chromium, µg/L	1.03 ± 0.44 [1758]	1.45 ± 0.40 [685]	<0.001
Copper, mg/L	0.93 ± 0.23^a^ [1798]	0.93 ± 0.21^a^ [685]	0.08
Iron, mg/L	1.31 ± 0.66 [1794]	1.17 ± 0.49 [687]	<0.001
Magnesium, mg/L	20.77 ± 2.56 [1791]	19.60 ± 2.02 [688]	<0.001
Manganese, µg/L	1.32 ± 0.73^a^ [1674]	1.34 ± 0.61^a^ [681]	0.96
Molybdenum, µg/L	1.05 ± 0.53 [1752]	1.34 ± 0.75 [682]	<0.001
Nickel, µg/L	1.18 ± 0.78 [1756]	2.07 ± 2.66 [685]	<0.001
Lead, µg/L	0.62 ± 1.14 [1765]	4.58 ± 6.79 [676]	<0.001
Rubidium, mg/L	0.41 ± 0.13 [1795]	0.38 ± 0.11 [688]	<0.001
Selenium, mg/L	0.16 ± 0.05 [1799]	0.12 ± 0.03 [688]	<0.001
Tin, µg/L	0.97 ± 1.05 [1793]	0.24 ± 0.21 [667]	<0.001
Strontium, µg/L	41.13 ± 18.47 [1797]	23.62 ± 6.91 [687]	<0.001
Titanium, µg/L	4.92 ± 3.34 [1796]	11.57 ± 3.16 [688]	<0.001
Vanadium, µg/L	3.25 ± 1.63 [1800]	2.15 ± 0.93 [688]	<0.001
Zinc, mg/L	0.85 ± 0.23 [1798]	1.30 ± 1.30 [684]	<0.001

Abbreviations: *FAMHES* the Fangchenggang Area Male Health and Examination Survey, *MEWHC* the manganese-exposed workers healthy cohort, *IQR* inter-quartile range, *BMI* body mass index.

Note: ^a^The metal concentration is not statistically different between the two cohorts; the number in square brackets represents the number of people finally included in the GWAS analysis for each metal.

### GWAS and meta-analysis

**Fig. 1:**
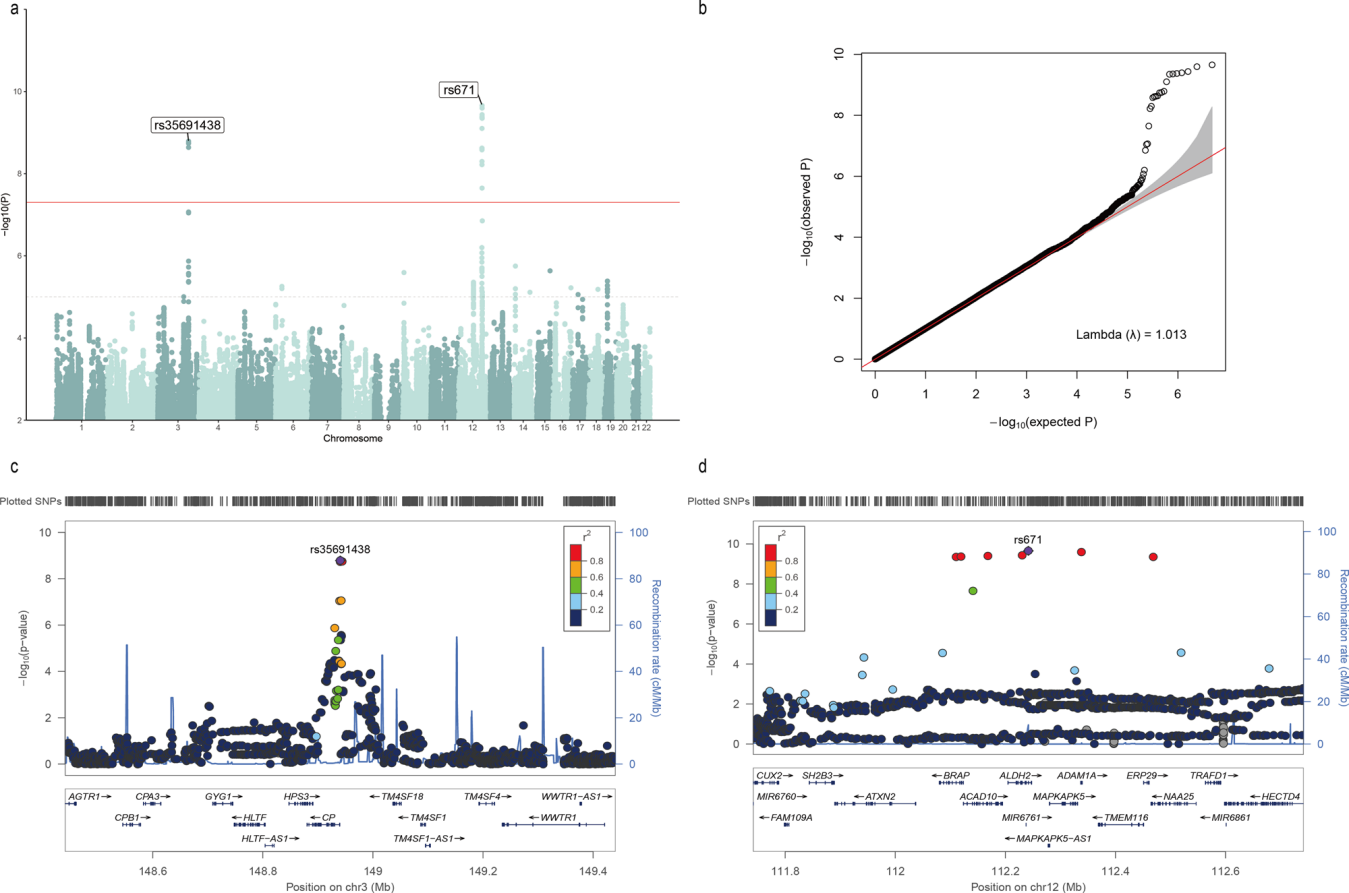
The Manhattan, Quantile-Quantile, and LocusZoom plots of GWAS meta-analysis of serum copper levels. **a** Manhattan plot. The *X*-axis presents the genomic position, while the *Y*-axis presents the −log_10_(*P*) of SNPs. The solid red line is the genome-wide significance threshold (*P* = 5.0 × 10^−8^), and the black dashed line is the genome-wide suggestive threshold (*P* = 1.0 × 10^−5^). **b** The Quantile-Quantile plot shows the deviation of the observed from the expected *P*-values under the null hypothesis of no association. **c** LocusZoom plot for gene *CP*. Purple diamond indicates SNP at the locus with the strongest association evidence (rs35691438) and the *r*^2^ (degree of linkage disequilibrium) is 1. Each point represents one SNP. **d** LocusZoom plot for gene *ALDH2*. Purple diamond indicates SNP at the locus with the strongest association evidence (rs671) and the *r*^2^ (degree of linkage disequilibrium) is 1.

We performed an independent GWAS for each transformed serum/plasma metal concentration in the Fangchenggang Area Male Health and Examination Survey (FAMHES) and the manganese-exposed workers healthy cohort (MEWHC), respectively. The specific number of subjects included in the GWAS of each metal is shown in Table 1. After imputing and quality control, the GWAS carried out in FAMHES and MEWHC included 7053840 and 4925397 variants, respectively. In FAMHES, two top SNPs (rs78069066, on *ADAM1A* of chromosome 12, *P* = 3.58 × 10^−9^; rs10424895, 0.901 kb away from *OR7A5* of chromosome 19, *P* = 4.42 × 10^−8^) were found significantly associated (*P* < 5.0 × 10^−8^) with serum copper levels (SCLs) (Supplementary Table 1). The Manhattan, Quantile-Quantile and LocusZoom plots are in Supplementary Fig. 1, and the genomic inflation factor (λ) is 1. In MEWHC, one SNP (rs2235321, on *TMPRSS6* of chromosome 22, *P* = 3.20 × 10^−9^) was found significantly associated with plasma iron levels (Supplementary Table 1). The Manhattan, Quantile-Quantile and LocusZoom plots are in Supplementary Fig. 2, and the genomic inflation factor (λ) is 1. For each metal, more information of SNPs that reached the suggestive threshold (*P* < 1.0 × 10^−5^) were in Supplementary Data 1. After stepwise conditional analyses, we didn’t find additional significant variants associated with SCLs in FAMHES and associated with plasma iron levels in MEWHC. For replication, we conducted a GWAS meta-analysis of serum/plasma copper levels using GWAS summary statistics from FAMHES and MEWHC. This meta-analysis was conducted using a fixed-effect inverse variance weighted method of PLINK 1.9. Two top SNPs (rs35691438, 0.84 kb away from *CP* of chromosome 3, *P*_meta_ = 1.63 × 10^−9^; rs671, on *ALDH2* of chromosome 12, *P*_meta_ = 2.21 × 10^−10^) were found significantly associated with transformed SCLs (Table 2). We also considered the Bonferroni-corrected threshold of <2.38 × 10^−9^ (5.0 × 10^−8^/(21 metals)), and both rs35691438 and rs671 still met the Bonferroni-corrected threshold. The Manhattan, Quantile-Quantile, and LocusZoom plots are in Fig. 1, and the genomic inflation factor (λ) is 1.013. Moreover, we found rs671 and rs78069066 are in extremely strong linkage disequilibrium (LD) with *r*^2^ = 0.99. We also conducted a GWAS meta-analysis of serum/plasma manganese level using GWAS summary statistics from FAMHES and MEWHC. However, no significant SNP was found.

**Table 2:** Top SNPs (*P* < 5E−8) associated with serum copper levels in the GWAS meta-analysis.

Metals	SNP	CHR	POS (hg19)	Gene	Function	EA	NEA	EAF	FAMHES				MEWHC				Meta (fixed-effect)					
									BETA	SE	*P*	*N*	BETA	SE	*P*	*N*	BETA	SE	*P*	*N*	*Q*	*I*^2^
SCLs	rs671	12	112241766	*ALDH2*	exonic	A	G	0.252	−0.226	0.038	4.52E−09	1798	−0.157	0.062	1.15E−02	685	−0.207	0.033	2.21E−10	2483	0.350	0
	rs35691438	3	148940672	*CP*(+0.84 kb)	upstream	C	T	0.325	0.175	0.035	8.29E−07	1798	0.225	0.064	4.65E−04	685	0.187	0.031	1.63E−09	2483	0.497	0

Abbreviations: *SCLs* serum copper levels, *SNP* single-nucleotide polymorphism, *CHR* chromosome, *Function* functional consequence on the gene, *POS* position, *EA* effect allele, *NEA* non-effect allele, *EAF* effect allele frequency, *N* the sample size of the genome-wide association studies or meta-analysis; *Q* *p*-value for Cochran’s *Q* statistic, *I*^2^ heterogeneity index (0−100 scale), *FAMHES* the Fangchenggang Area Male Health and Examination Survey, *MEWHC* the manganese-exposed workers healthy cohort, *Meta* meta-analysis of FAMHES and MEWHC.

### Post-GWAS analysis

Four significant independent SNPs (*r*^2^ < 0.6) were identified from the GWAS meta-analysis of SCLs via Functional Mapping and Annotation (FUMA) (Supplementary Table 2). A total of 16 potential causal genes involved in SCLs were identified via positional mapping and eQTL (expression quantitative trait loci) mapping of FUMA (Supplementary Data 2). Through gene-based analysis, we found two genes (*CP* on chromosome 3, *P* = 1.94 × 10^−6^; *RPH3A* on chromosome 12, *P* = 2.50 × 10^−6^) reached the significance threshold (*P* = 0.05/17322 = 2.887 × 10^−6^). The number 17322 is the quantity of protein-coding genes which are mapped based on the GWAS meta-analysis summary statistics. More information for the first 30 genes with the lowest *P*-value of gene-based analysis were in Supplementary Table 3. We didn’t find any significant (*P* < 0.05/15482 = 3.2296 × 10^−6^) gene-set via gene-set analysis. The number 15482 is the quantity of gene sets. More information for the first 30 gene-sets with the lowest *P*-value of gene-set analysis were in Supplementary Table 4. We used the significant genes from gene-based analysis and mapped genes to construct the gene expression heatmap; it shows in Supplementary Fig. 3.

### MR

**Fig. 2:**
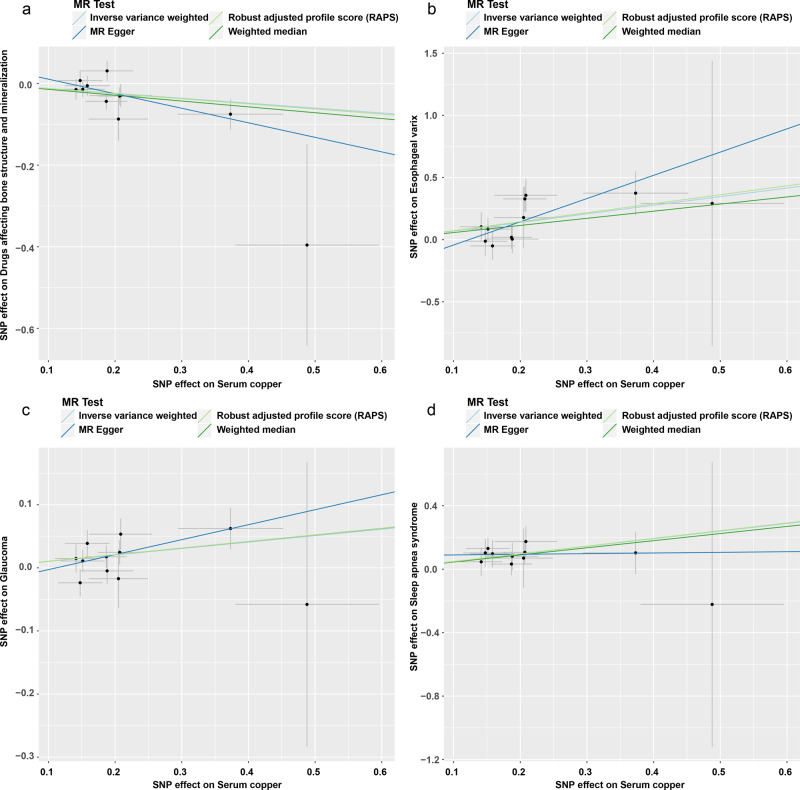
Scatter plots for four robust results in the first round two-sample Mendelian randomization analyses. The error bars indicate standard error. **a** Usage of drugs affecting bone structure and mineralization. **b** Esophageal varix. **c** Glaucoma. **d** Sleep apnea syndrome. MR Mendelian randomization.

**Fig. 3:**
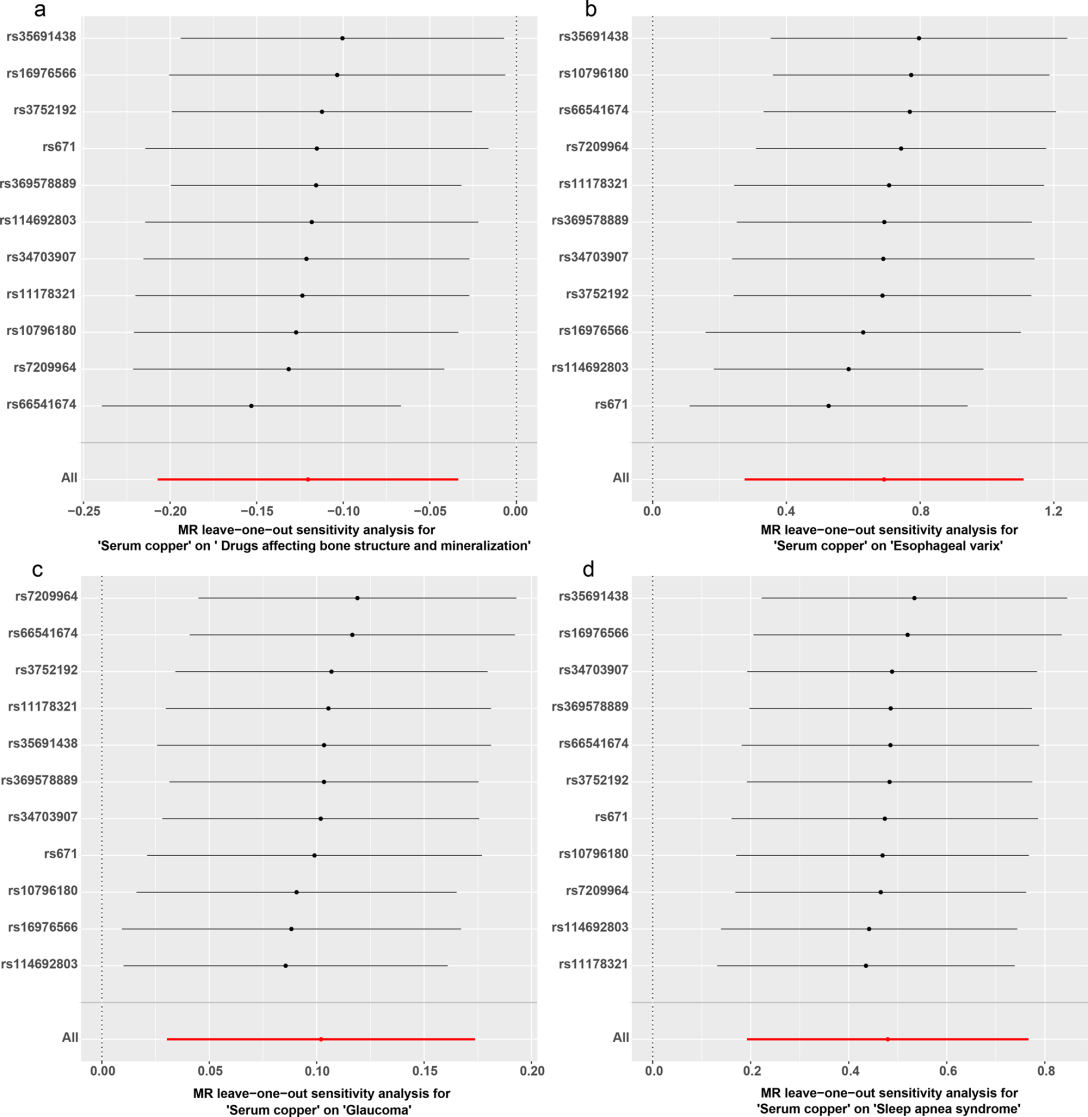
Plots of leave-one-out analyses for four robust results in the first round two-sample Mendelian randomization analyses. The error bars indicate the 95% confidence interval. **a** Usage of drugs affecting bone structure and mineralization. **b** Esophageal varix. **c** Glaucoma. **d** Sleep apnea syndrome. MR Mendelian randomization.

**Fig. 4:**
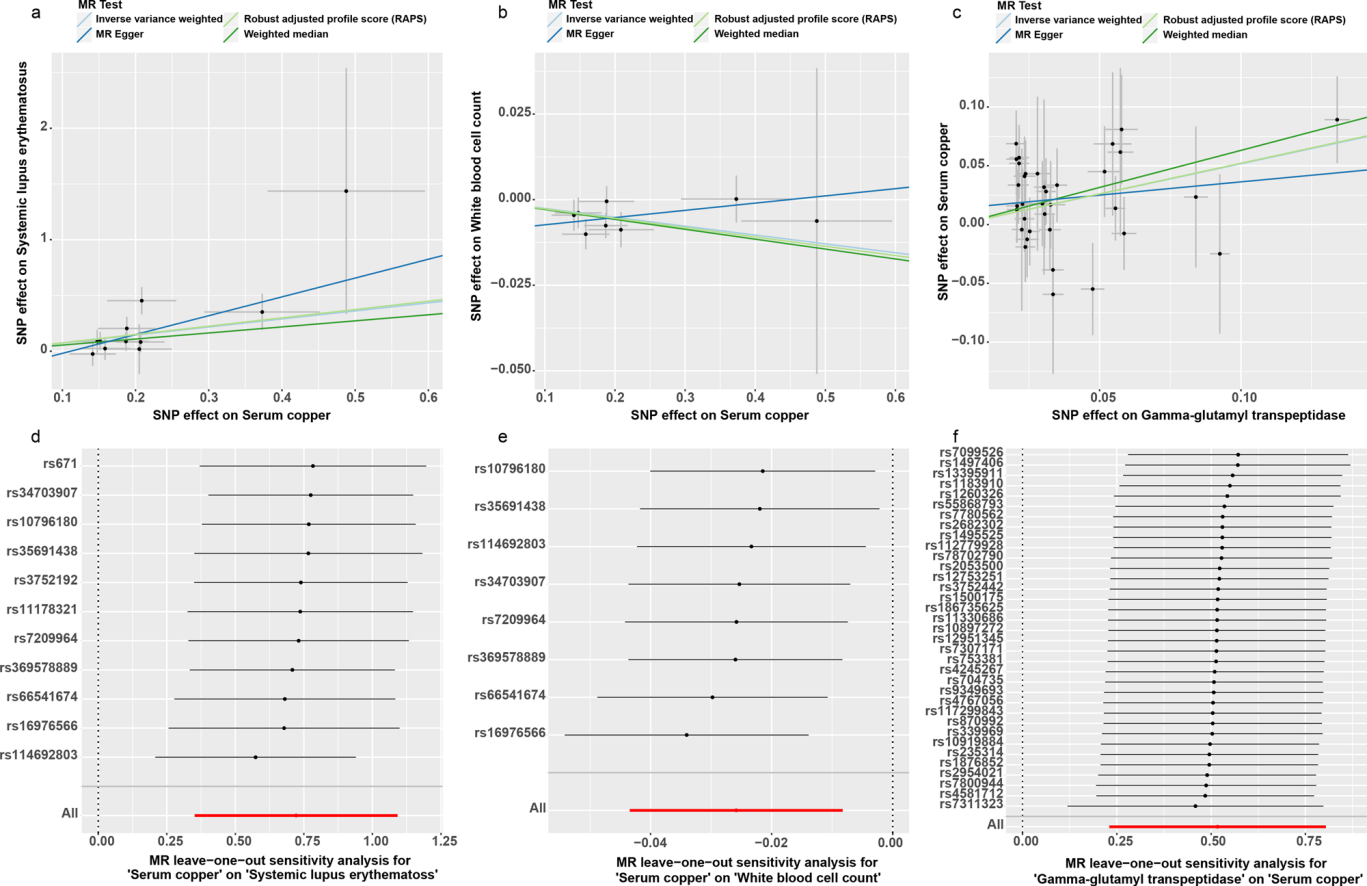
Scatter and leave-one-out analyses plots for two robust results in the first round two-sample Mendelian randomization analyses and one robust result in the second round two-sample Mendelian randomization analyses. The error bars in panels **a**–**c** indicate standard error. The error bars in panels **d**–**f** indicate the 95% confidence interval. **a**, **d** Systemic lupus erythematosus. **b**, **e** White blood cell count. **c**, **f** Gamma-glutamyl transpeptidase levels are exposure factors, and serum copper levels are the outcome. MR Mendelian randomization.

In the first round of two-sample MR in East Asian population, we investigated the causal effects of SCLs on 213 deep phenotypes. Firstly, we found 21 exposure-outcome pairs might have causal relationships (Supplementary Table 5). Secondly, for the pairs which had significant horizontal pleiotropy or heterogeneity, we re-performed two-sample MR analyses after excluding the outliers (*P*-value < 0.05) identified in the Mendelian randomization pleiotropy residual sum and outlier (MR-PRESSO) outlier test. Finally, six exposure-outcome pairs which could draw robust causal conclusions were put into Table 3, including 1 medication usage outcome: drugs affecting bone structure and mineralization (ATC code M05B) (inverse-variance weighted [IVW]: odds ratio [OR] 95% confidence interval [95% CI] 0.887 (0.813 0.967), *P* = 0.007, Power = 0.83; weighted median [WM]: OR (95% CI) 0.867 (0.771 0.974), *P* = 0.016, Power = 0.93; MR using the robust adjusted profile score [MR-RAPS]: OR (95% CI) 0.883 (0.809 0.963), *P* = 0.005, Power = 0.85); and 4 disease outcomes: esophageal varix (IVW: OR (95% CI) 1.998 (1.317 3.032), *P* = 0.001, Power = 1.00; MR-Egger: OR (95% CI) 6.479 (1.394 30.108), *P* = 0.041, Power = 1.00; WM: OR (95% CI) 1.773 (1.001 3.141), *P* = 0.0497, Power = 0.99; MR-RAPS: OR (95% CI) 2.060 (1.372 3.093), *P* = 0.0005, Power = 1.00); glaucoma (IVW: OR (95% CI) 1.107 (1.031 1.190), *P* = 0.005, Power = 0.89; WM: OR (95% CI) 1.110 (1.009 1.222), *P* = 0.032, Power = 0.90; MR-RAPS: OR (95% CI) 1.110 (1.029 1.198), *P* = 0.007, Power = 0.90); Sleep apnea syndrome (IVW: OR (95% CI) 1.615 (1.212 2.153), *P* = 0.001, Power = 0.99; WM: OR (95% CI) 1.567 (1.078 2.276), *P* = 0.019, Power = 0.98; MR-RAPS: OR (95% CI) 1.625 (1.191 2.218), *P* = 0.002, Power = 0.99); Systemic lupus erythematosus (SLE) (IVW: OR (95% CI) 2.058 (1.422 2.980), *P* = 0.0001, Power = 1.00; MR-Egger: OR (95% CI) 5.412 (1.344 21.788), *P* = 0.041, Power = 1.00; WM: OR (95% CI) 1.722 (1.070 2.770), *P* = 0.025, Power = 0.99; MR-RAPS: OR (95% CI) 2.110 (1.451 3.068), *P* = 0.00009, Power = 1.00); and 1 biomarker outcome: white blood cell (WBC) count (IVW: β (95% CI) −0.026 (−0.043 −0.008), *P* = 0.004; WM: β (95% CI) −0.029 (−0.053 −0.005), *P* = 0.020; MR-RAPS: β (95% CI) −0.027 (−0.046 −0.008), *P* = 0.005). The results of IVW and MR-RAPS methods for SLE are still significant with the corresponding *P*-value smaller than the Bonferroni-corrected threshold (0.00023); however, the results of IVW, WM and MR-RAPS methods for the other five exposure-outcome pairs and the results of MR-Egger method for esophageal varix and SLE are suggestively significant with the corresponding *P*-value smaller than 0.05 but larger than the Bonferroni-corrected threshold (0.00023). Their scatter plots and plots of leave-one-out analyses were displayed in Figs. 2–4. Thirteen exposure-outcome pairs which could not draw robust causal conclusions were put into Supplementary Table 6, and their plots of leave-one-out analysis were in Supplementary Figs. 4−7.

**Table 3:** Significant robust results of the first and second round two-sample Mendelian randomization analyses.

Exposure	Outcome	SNPs	IVW					MR-Egger							WM			MR-RAPS		
			OR (95% CI)	*P* value	Power	Cochran Q statistics (df)	*P* value	OR (95% CI)	*P* value	Power	Intercept (Se)	*P* value	Cochran Q statistics (df)	*P* value	OR (95% CI)	*P* value	Power	OR (95% CI)	*P* value	Power
SCLs	Drugs affecting bone structure and mineralization	11	0.887 (0.813 0.967)	0.007	0.83	11.275 (10)	0.336	0.700 (0.509 0.963)	0.056	1.00	0.046 (0.031)	0.168	9.021 (9)	0.435	0.867 (0.771 0.974)	0.016	0.93	0.883 (0.809 0.963)	0.005	0.85
	Esophageal varix	11	1.998 (1.317 3.032)	0.001	1.00	11.948 (10)	0.289	6.479 (1.394 30.108)	0.041	1.00	−0.230 (0.148)	0.155	9.427 (9)	0.399	1.773 (1.001 3.141)	0.0497	0.99	2.060 (1.372 3.093)	0.0005	1.00
	Glaucoma	11	1.107 (1.031 1.190)	0.005	0.89	8.752 (10)	0.556	1.268 (0.959 1.675)	0.130	1.00	−0.027 (0.027)	0.351	7.784 (9)	0.556	1.110 (1.009 1.222)	0.032	0.90	1.110 (1.029 1.198)	0.007	0.90
	Sleep apnea syndrome	11	1.615 (1.212 2.153)	0.001	0.99	2.815 (10)	0.985	1.042 (0.335 3.241)	0.945	0.06	0.086 (0.109)	0.454	2.202 (9)	0.988	1.567 (1.078 2.276)	0.019	0.98	1.625 (1.191 2.218)	0.002	0.99
	Systemic lupus erythematosus	11	2.058 (1.422 2.980)	0.0001	1.00	11.266 (10)	0.337	5.412 (1.344 21.788)	0.041	1.00	−0.189 (0.134)	0.193	9.235 (9)	0.416	1.722 (1.070 2.770)	0.025	0.99	2.110 (1.451 3.068)	0.00009	1.00
	White blood cell count (WBC)^a^	8	−0.026 (−0.043 −0.008)	0.004	—	5.921 (7)	0.549	0.021 (-0.041 0.083)	0.527	—	−0.009 (0.006)	0.170	3.492 (6)	0.745	−0.029 (−0.053 −0.005)	0.020	—	−0.027 (−0.046 −0.008)	0.005	—
Gamma-glutamyl transpeptidase (GGT) levels^a^	SCLs	35	0.517 (0.230 0.804)	0.0004	—	30.822 (34)	0.624	0.226 (-0.316 0.767)	0.420	—	0.014 (0.011)	0.222	29.273 (33)	0.653	0.631 (0.194 1.068)	0.005	—	0.491 (0.210 0.772)	0.0006	—

Abbreviations: *SCLs* serum copper levels, *MR* Mendelian randomization, *SNPs* number of single-nucleotide polymorphism used as instrumental variables, *IVW* inverse-variance weighted, *WM* weighted median, *MR-RAPS* Mendelian randomization using the robust adjusted profile score, *OR* odds ratio.

Note: ^a^For each method, we give β and 95% confidence interval instead of OR and 95% confidence interval.

In the second round of two-sample MR in the East Asian population, we investigated the causal effects of 213 deep phenotypes on SCLs. Firstly, we found 16 exposure-outcome pairs might have causal relationships (Supplementary Table 7). Secondly, we re-performed the two-sample MR analysis after excluding the outliers (*P-*value < 0.05) identified in the MR-PRESSO outlier test for the pairs with significant horizontal pleiotropy or heterogeneity. Finally, one exposure-outcome pair which could draw a robust causal conclusion was put into Table 3. It shows that genetically predicted gamma-glutamyl transpeptidase (GGT) is positively associated with SCLs (IVW: β (95% CI) 0.517 (0.230 0.804), *P* = 0.0004; WM: β (95% CI) 0.631 (0.194 1.068), *P* = 0.005; MR-RAPS: β (95% CI) 0.491 (0.210 0.772), *P* = 0.0006). The results of IVW, WM and MR-RAPS methods for this exposure-outcome pair are suggestively significant with the corresponding *P*-value smaller than 0.05 but larger than the Bonferroni-corrected threshold (0.00023). Its scatter plot and plot of leave-one-out analysis are displayed in Fig. 4. The information of genetic instrumental variables (IVs) of GGT levels is shown in Supplementary Table 8. Eight exposure-outcome pairs which could not draw robust causal conclusions were put into Supplementary Table 9, and their plots of leave-one-out analysis were in Supplementary Figs. 8, 9.

**Fig. 5:**
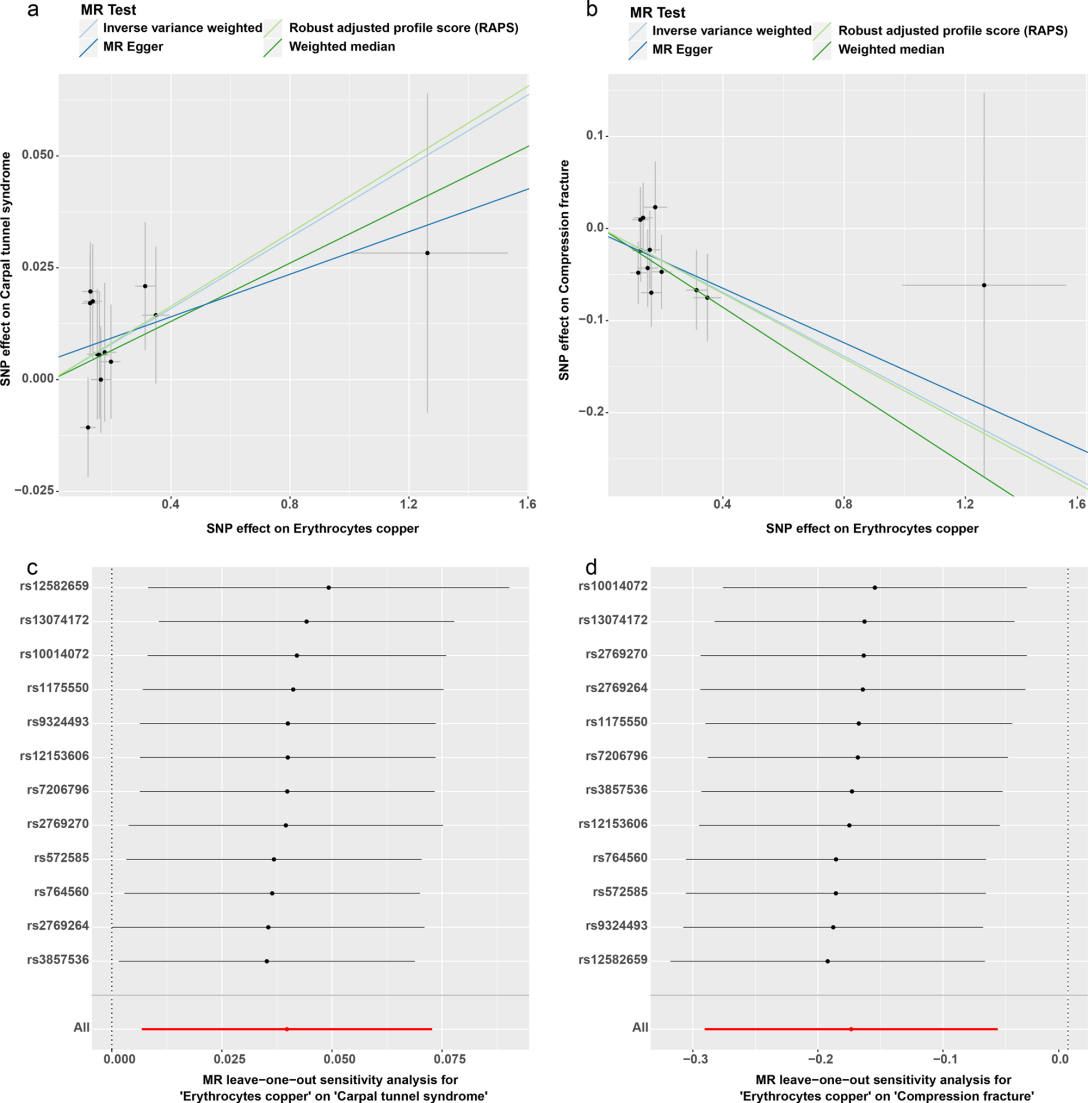
Scatter and leave-one-out analyses plots for two robust results in the third round two-sample Mendelian randomization analyses. The error bars in panels **a** and **b** indicate standard error. The error bars in panels **c** and **d** indicate the 95% confidence interval. **a**, **c** Carpal tunnel syndrome. **b**, **d** Compression fracture. MR Mendelian randomization.

In the third round of two-sample MR in the European population, we investigated the causal effects of erythrocytes copper levels (ECLs) on 174 deep phenotypes. Firstly, we found 16 exposure-outcome pairs might have causal relationships (Supplementary Table 10). Secondly, for the pairs which have significant horizontal pleiotropy or heterogeneity, we re-performed two-sample MR analyses after excluding the outliers (*P*-value < 0.05) identified in the MR-PRESSO outlier test. Finally, two exposure-outcome pairs which could draw robust causal conclusions were put into Table 4, including 2 disease outcomes: carpal tunnel syndrome (IVW: OR (95% CI) 1.041 (1.007 1.075), *P* = 0.018, Power = 0.62; MR-RAPS: OR (95% CI) 1.042 (1.005 1.080), *P* = 0.024, Power = 0.64); compression fracture (IVW: OR (95% CI) 0.841 (0.748 0.945), *P* = 0.004, Power = 0.79; WM: OR (95% CI) 0.807 (0.693 0.941), *P* = 0.006, Power = 0.92; MR-RAPS: OR (95% CI) 0.838 (0.740 0.949), *P* = 0.005, Power = 0.81). The results of IVW, WM, and MR-RAPS methods for compression fracture are suggestively significant with the corresponding *P*-value smaller than 0.05 but larger than the Bonferroni-corrected threshold (0.00023). The results of IVW and MR-RAPS methods for carpal tunnel syndrome are suggestively significant with the corresponding *P*-value smaller than 0.05 but larger than the Bonferroni-corrected threshold (0.00023). Their scatter plots and plots of leave-one-out analyses were in Fig. 5. Fourteen exposure-outcome pairs which could not draw robust causal conclusions were put into Supplementary Table 11, and their plots of leave-one-out analysis were in Supplementary Figs. 10–13.

**Table 4:** Significant robust results of the third round two-sample Mendelian randomization analyses.

Exposure	Outcome	SNPs	IVW					MR-Egger							WM			MR-RAPS		
			OR (95% CI)	*P* value	Power	Cochran *Q* statistics (df)	*P* value	OR (95% CI)	*P* value	Power	Intercept (Se)	*P* value	Cochran *Q* statistics (df)	*P* value	OR (95% CI)	*P* value	Power	OR (95% CI)	*P* value	Power
ECLs	Carpal tunnel syndrome	12	1.041 (1.007 1.075)	0.018	0.62	6.792 (11)	0.816	1.024 (0.968 1.083)	0.426	0.27	0.005 (0.007)	0.507	6.318 (10)	0.788	1.033 (0.988 1.080)	0.153	0.45	1.042 (1.005 1.080)	0.024	0.64
	Compression fracture	12	0.841 (0.748 0.945)	0.004	0.79	5.706 (11)	0.892	0.862 (0.668 1.113)	0.283	0.67	−0.006 (0.026)	0.831	5.658 (10)	0.843	0.807 (0.693 0.941)	0.006	0.92	0.838 (0.740 0.949)	0.005	0.81

Abbreviations: *ECLs* erythrocytes copper levels, *MR* Mendelian randomization, *SNPs* number of single-nucleotide polymorphism used as instrumental variables, *IVW* inverse-variance weighted, *WM* weighted median, *MR-RAPS* Mendelian randomization using the robust adjusted profile score, *OR* odds ratio.

## Discussion

We identified two SNPs (rs35691438 and rs671) that were significantly associated with SCLs in the Chinese population. In the East Asian population, GGT levels had a causal effect on the SCLs, and SCLs have causal effects on six outcomes, namely risks of esophageal varix, glaucoma, sleep apnea syndrome, and SLE, WBC count, and usage of drugs affecting bone structure and mineralization. In the European population, ECLs have causal effects on risks of carpal tunnel syndrome and compression fracture.

We found that SNP rs671 was significantly associated with SCLs. Many studies have reported associations between rs671 and other phenotypes, such as alcohol consumption (drinkers vs non-drinkers), coffee consumption, serum alpha1-antitrypsin levels, esophageal cancer, gout^21–25^. SNP rs671 is a non-synonymous SNP within the exonic region of *ALDH2*, which is widely expressed in many human tissues, especially fat and liver. The encoded ALDH2 protein belongs to the aldehyde dehydrogenase family and is involved in the synthesis of acetate from ethanol in the alcohol metabolism pathway. Though there are some researchers reported links between SCLs and alcohol consumption^26–30^, it still needs further study to clarify the intricate relationship, such as the potential association between rs671 and SCLs may be mediated by alcohol drinking behavior, between SCLs and alcohol consumption behavior on various metabolic conditions^31–33^. For rs35691438, which was significantly associated with SCLs in our study, we found no reports of associations between it and other phenotypes. The *CP* gene, which is close to rs35691438, is highly expressed mainly in the liver. *CP* encodes ceruloplasmin, a metalloprotein that binds most of the copper in plasma and is involved in the peroxidation of Fe (II) transferrin to Fe (III) transferrin^34,35^. Mutations in *CP* cause aceruloplasminemia, which results in iron accumulation and tissue damage and is associated with diabetes and neurologic abnormalities. To date, a study reported 2 SNPs (rs1175550 and rs2769264) on chromosome 1 significantly (*P* < 5.0 × 10^−8^) associated with ECLs^6^, whereas another study found no SNPs that were associated with whole blood copper levels (BCLs) at the significance threshold (*P* < 5.0 × 10^−8^)^9^. The finding of Evans et al.^6^ has not been replicated in other studies. In our GWAS, we originally planned to test two SNPs (rs1175550 and rs2769264) on chromosome 1. However, because rs1175550 had a minor allele frequency (MAF) of < 0.01 in the East Asian population and was excluded according to the quality control standards, rs1175550 was not included in the GWAS. Although rs2769264 was found in our SCLs GWAS meta-analysis results, it was not significant (*P* = 0.4646); the significance threshold was *P* < 1.0 × 10^−5^. We found that the MAF of rs1175550 varied widely between the East Asian and European populations in the 1000 Genomes project (http://www.1000genomes.org); 0.2% in East Asians and 21.9% in Europeans. The MAF of rs2769264 also varied widely; 28.6% in East Asians and 16.1% in Europeans. These inter-ethnic discrepancies in the MAFs and the differences between disparate biological samples (serum copper and erythrocytes copper) may explain the highly significant association of rs2769264 in Europeans and no significant association in East Asians^36^. Moreover, rs2235321, a synonymous SNP on the transmembrane serine protease 6 (*TMPRSS6*) gene of chromosome 22, was significantly associated with plasma iron levels in MEWHC. A previous GWAS reported five SNPs (rs8177240, rs1800562, rs1799945, rs7385804, rs855791) that were significantly associated with serum iron levels^7^. We checked these five SNPs in the results of our GWAS of the MEWHC and found that only rs855791 (in *TMPRSS6* in strong LD (*r*^2^ = 0.90) with rs2235321) was significant (*P* = 1.19 × 10^−8^). The number of individuals (687) in our study is quite different from the number (48972) in the study of Benyamin et al.^7^, which may explain why the other four SNPs were not significant in our study.

In the first round of two-sample MR analyses conducted in the East Asian population, we found that genetically higher SCLs had causal effects on lower trends for the usage of drugs affecting bone structure and mineralization (ATC code M05B), fewer counts of WBC. The genetically increased SCLs also have causal effects on higher risks of esophageal varix, glaucoma, sleep apnea syndrome, and SLE. No direct study of the relationship between SCLs and the usage of M05B Drugs has been reported so far. M05B Drugs include mainly M05BA (bisphosphonates), M05BB (bisphosphonates, combinations), and M05BC (bone morphogenetic proteins). The bisphosphonates and bone morphogenetic proteins are both conducive to bone formation^37–39^. Animal studies have shown that copper deficiency decreased bone strength^40^, and that the released copper stimulated bone formation^41^. Our results are indirectly consistent with the results of these studies.

Esophageal varix is related to cirrhosis and portal hypertension^42^. Until now, seldom studies have directly focused on the link between SCLs and esophageal varix. However, numerous studies have shown that the accumulation of copper can lead to serious liver diseases, including hepatitis and cirrhosis, whether in Wilson’s disease or a non-Wilson’s disease^43–48^. One epidemiological study found that SCLs were increased in patients with liver cirrhosis and variceal bleeding and suggested that variceal bleeding in patients with liver cirrhosis was associated with important imbalances in blood concentrations of zinc, copper, and magnesium^49^. Although the specific biological mechanism between increased SCLs and the higher risk of esophageal varix is unclear, our MR finding is consistent with what was reported by Dumea et al.^49^ and further showed the possible causal relationship between them. Therefore, we speculate that esophageal varix may be triggered by cirrhosis, which is caused by the toxicity of copper. An in-depth study of the mechanism needs to be carried out in future work.

Glaucoma is a complex disease with the main risk factor being intraocular pressure. However, previous studies suggested that the further pathogenic factors in glaucoma should include oxidative stress. Oxidative stress can destroy neurons and alter the structure of the trabecular meshwork, which is the principal out channel of aqueous humor, while intraocular pressure continuously increases^50,51^. A study showed that unbalanced copper levels could lead to oxidative stress through the reduction of glutathione or the Fenton reaction. In a state of oxidative stress, large numbers of reactive oxygen species are generated, which induces subsequent lipid peroxidation and DNA damage^52^. An early epidemiological study reported that higher mean SCLs were seen in the patients with glaucoma^53^. Another study showed that the SCLs increased in females with primary open-angle glaucoma compared with that of males with or without primary open-angle glaucoma^54^. Therefore, copper ions may mediate the occurrence of glaucoma via oxidative stress. Our results are consistent with those of previous studies.

Sleep apnea is a condition in which patients have repeated bouts of shallow breathing or apnea while sleeping. It is related to oxidative stress, high blood pressure, hypercoagulation, and inflammation, and can cause episodic hypoxemia^55–57^. Although copper is closely related to the oxidative stress state of the human body, no previous epidemiological studies have reported associations between SCLs and sleep apnea syndrome. Only one study compared complaints and disease symptoms of obstructive sleep apnea syndrome in patients with Wilson’s disease, which resulted in copper overload, and healthy controls; no significant difference was found between them^58^. Conversely, we found a strong causal effect of SCLs on sleep apnea syndrome, which suggests this relationship is well worth exploring in future studies.

SLE is an inflammatory autoimmune disease in which the immune system destroys healthy cells and tissues throughout the body^59,60^. Higher SCLs have been found in patients with SLE^61–63^. However, another study reported that there was no significant difference in SCLs between SLE patients and healthy controls^64^. The results of traditional epidemiological studies on the relationship between SCLs and SLE disease activity are controversial. Yilmaz et al. found that there was a positive correlation between SCLs and SLE disease activity^63^, but Sahebari et al. reported that there was a negative correlation between SCLs and SLE disease activity^64^. Ferns et al. proposed that copper had both pro- and anti-oxidative in vivo effect^65^. With regard to the pathogenic effect of reactive oxygen species on SLE, previous studies generally believed that reactive oxygen species is an important pathogenic factor, but recent studies believe that insufficient production of reactive oxygen species will lead to the occurrence of SLE^66,67^. There is a sharp contrast between these research results. Our research provides clues to the current controversial epidemiological results from a genetic point of view, and further corresponding research is needed in the future.

Although no epidemiological research on the association between SCLs and WBC count has been reported, we found some definitive evidence of such an association. Serum copper and zinc concentrations were found to be higher in users of proton pump inhibitors than in controls, and WBC counts may reduce with long‑term proton pump inhibitor therapy^68^. Copper ions have been shown to aid in the regulation of disulfiram cytotoxicity in myeloid leukemia cells. Copper can help patients with high WBC counts by restoring the sensitivity of high-density leukemic cells to disulfiram and improving the efficacy of anti-leukemic therapies that include disulfiram^69^. Further research on this relationship between SCLs and WBC count is well worth exploring.

To fully understand whether these 213 deep phenotypes have causal effects on SCLs, we conducted the second round of two-sample MR analyses in the East Asian population. We found that genetically high GGT levels have a causal effect on elevated SCLs. GGT is a hepatic enzyme that aids in the production of the antioxidant glutathione at the outer surface of the plasma membrane^70^. In clinical laboratories, serum GGT has long been used as a marker for excessive alcohol intake and obesity, particularly in nonalcoholic fatty liver disease, alcoholic liver disease, and chronic hepatitis^71^. Indeed, serum GGT is a very early and sensitive indicator of oxidative stress and inflammation^72^. One epidemiological study suggested that serum GGT levels were positively correlated with SCLs^73^. Our study is the first to describe the causal relationship between them. Although both copper and GGT are involved in oxidative stress and inflammation^74,75^, the mechanism of the causal relationship between them has not been reported so far. The specific biological mechanism is well worth discovering.

On the basis of our robust first and second round MR results for the East Asians, we inferred that serum copper might have a mediation effect on the causal effects of the GGT levels on the risk of esophageal varix, glaucoma, sleep apnea syndrome, and SLE, WBC count, and the possibility of using drugs affecting bone structure and mineralization. Serum GGT levels have been reported to be associated with the severity of obstructive sleep apnea syndrome^76,77^. A recent case-control study found that the risk of SLE increased with increased GGT levels^78^. Another early study reported that an increase of urinary GGT activity was seen in patients with SLE^79^. A study of Indian children and another study of Korean adults both showed that GGT was positively associated with WBC count^80,81^. These studies showed associations between GGT levels and sleep apnea syndrome, SLE, and WBC count. We did not find relevant literature for the other diseases and traits. Currently, there are no MR studies that have directly investigated causality between GGT levels and these six phenotypes. Because of the limitation of data acquisition, we did not conduct MR analysis on these possible relationships, and did not deeply explore whether serum copper might play a mediating role or determine the extent of the possible mediating effect of serum copper in these processes. Further independent studies aimed at solving these problems are needed.

To further study the causal effects of BCLs on these deep phenotypes of Europeans, we conducted a third round of two-sample MR analyses in European populations. We found that genetically high ECLs had causal effects on the higher risk of carpal tunnel syndrome and lower risk of compression fracture.

The most common nerve entrapment syndrome is carpal tunnel syndrome, and it has been studied extensively^82^. Carpal tunnel syndrome is produced when the median nerve in the wrist was compressed while passing through a space-constrained osteofibrous canal^83^. Although no epidemiological study of the association between ECLs and carpal tunnel syndrome has been reported so far, our results provide valuable evidence that is worth exploring. The causal effect of copper on carpal tunnel syndrome be based on the neurotoxicity of copper ions and the oxidative stress induced by copper. To corroborate this relationship, independent cohort and biological function studies are needed.

So far, we have not found epidemiological studies of ECLs and compression fracture. However, many studies have shown that copper plays a vital role in maintaining bone health. Copper eliminates bone-free radicals generated by osteoclast activity as a cofactor in antioxidant enzymes^84^, and copper has been suggested to suppress osteoclastic resorption directly^85^. Conversely, a national cross-sectional study in the United States suggested that high SCLs were strongly related to increased total fracture^86^. The differences among these studies can be attributed to the different ethnicities, sample types, and other possible confounding factors that were not controlled. Our finding that genetically high ECLs were protective against the risk of compression fracture provides a reference for subsequent epidemiological studies and a more in-depth analysis of the biological mechanisms involved.

Interestingly, we found that two diseases, food allergy, and urticaria, both appeared in the European and East Asian MR results where robust conclusions could not be drawn. Although our results did not show a robust causal relationship between these diseases and BCLs, our research gives suggestive clues on the causal relationship between them. Moreover, we found that SLE and the usage of drugs affecting bone structure and mineralization both appeared in the robust results of the East Asian MR and results where we could not draw robust conclusions of the European MR analyses. We also found that compression fracture appeared in the robust results of European MR and results where we could not draw robust conclusions of East Asian MR analyses. Because we used genetic IVs related to SCLs for the East Asian MR analyses and genetic IVs related to ECLs for the European MR analyses, whether differences of significance for the same disease in different populations can be attributed to ethnic differences needs to be studied further.

We noted that another BCLs MR analysis from UK Biobank indicated that genetically increased ECLs were causally associated with a reduced risk of iron deficiency anemia, whereas this association was not significant in our study^18^. We can suggest two possible explanations for this difference. (1) We used the GWAS meta-analysis summary statistics of UK Biobank and FinnGen for iron deficiency anemia, which increased the number of cases compared with the number for the single GWAS summary statistic from UK Biobank or FinnGen. However, because using the GWAS meta-analysis summary statistics may also greatly increase the overall number, the proportion of cases in the total number for a specific disease may decrease. Such decreased proportions will lead to weaker statistical power, which may have led to our failure to detect a causal effect between iron deficiency anemia and ECLs. (2) In their study, Zhou et al. used only two IVs that were associated with ECLs, and then conducted a PheWAS-MR (phenome-wide association study and MR) analysis. After the PheWAS, some phenotypes unrelated to the selected genetic variants were excluded, and their MR analysis did not include them. However, we included 12 SNPs as IVs in the MR analysis, which increased the statistical power. This may explain the difference to a certain extent. Our significantly robust results were not referred to in their paper.

To the best of our knowledge, ours is the first reported systematic GWAS of 21 BMLs using two independent prospective East Asian cohorts. Compared with the GWAS of 11 BMLs conducted in the European population by Ng et al.^9^, we have included more metals and discovered loci for SCLs. To our knowledge, this may also be the first study to use bidirectional two-sample MR analysis with hypothesis-free investigations of the causal effects between BCLs and 213 deep phenotypes in the East Asian population. We also used two-sample MR analysis with hypothesis-free investigations of the causal effects of BCLs on 174 deep phenotypes in the European population. This approach provides a more comprehensive understanding of the impact of BCLs on the health of East Asians and Europeans. Moreover, the impacts of potential weak instrumental variable bias, heterogeneity, and pleiotropic effects on the results were also considered. Possible biases caused by horizontal pleiotropy were fully taken into account in our analyses process. We used the MR-PRESSO outlier test and other robust methods for the MR analysis, and no horizontal pleiotropy was detected in the results. The PhenoScanner database or the Ensembl Project would not necessarily distinguish between vertical and horizontal pleiotropy, and the former would not bias the MR analysis^87^. Furthermore, because the biological mechanisms and functions of numerous genetic variants are unknown, we did not use these variants to assess the IVs associated with potential confounding factors. The genetic IVs we used explain 11.09% of the variance in SCLs and 17.06% of the variance in ECLs, which increased the statistical power to identify significant associations^88^. F statistics were considered to avoid weak instrument bias, and *I*^2^, which evaluates the heterogeneity scale of IVs, was used to reduce the impact of potential heterogeneity. The presence of horizontal pleiotropy was examined or corrected using MR-Egger tests and MR-PRESSO. We used the leave-one-out sensitivity analysis to ensure no results were impacted by specific variants.

Our study has several limitations. Although we conducted the GWAS on 21 serum and plasma metals, because of differences in the concentrations of the same metals in serum and plasma, we only did the meta-analyses of the serum copper and serum manganese. The GWASs of the remaining 19 serum/plasma metals need to be combined with other appropriate independent cohorts for further replication in the future. Because relatively small numbers of cases were used in the GWAS, it was difficult to find significant SNPs. Despite this, we found loci for SCLs; however, more research is needed to verify our conclusions. In the two-sample MR analysis, we did not find genetic IVs of ECLs or SCLs in the East Asian and European populations, respectively, which limited our comprehensive study of the causal effects of BCLs on 213 deep phenotypes in these populations. For the same reason, we separately included the genetic IVs related to the SCLs of East Asians and the ECLs of Europeans, which had an impact on the horizontal comparison of differences in the same exposure between ethnicities. Although approximately 800,000 individuals were included in the MR analyses, the number of cases of specific diseases was still small, which reduced the statistical power. Moreover, because access to data was limited, our MR results were not verified in large independent databases.

## Methods

### Subjects

To conduct comprehensive GWASs of 22 serum/plasma metal levels in the Chinese population, we used the serum metal levels data obtained from FAMHES in Guangxi Fangchenggang and plasma metal levels data obtained from MEWHC. FAMHES included 4303 Chinese males aged 17–88 years old at baseline. Their questionnaires and physical examinations at baseline were completed in the Medical Center of Fangchenggang First People’s Hospital between September 2009 and December 2009^89^. MEWHC included 825 Chinese with their follow-up completed in 2017. More detailed information about FAMHES, MEWHC were introduced elsewhere^90,91^. Our study was approved by the Ethics and Human Subject Committee of Guangxi Medical University. All the subjects provided written informed consent before participating in the study.

### Phenotypes

All blood samples are stored at −80 °C until analysis. We used an inductively coupled plasma mass spectrometry (ICP-MS, Perkin Elmer NexION 350xX, USA) to measure the serum metal concentrations of FAMHES and plasma metal concentrations of MEWHC, respectively. Our previous research also introduced the corresponding methods^89,92^. Specifically, each serum/plasma sample was diluted 20 times by using acidic solution containing 0.01% Triton^TM^ X-100, 0.5% n-butyl alcohol and 1% nitric acid. We used ClinChek^®^ human serum controls for trace elements (order no.8880 and order no.8881, Recipe Chemicals, Germany) and Standard Reference Material^®^1640a (Trace Elements in Natural Water, National Institute of Standards and Technology, USA) as standard reagent for quality assurance in serum measurement. Moreover, we used ClinChek^®^ human plasma controls for trace elements (order no.8,885; Recipe Chemicals, Germany) and Standard Reference Material^®^1640a for quality assurance in plasma measurement. The following 22 metals (aluminum, antimony, arsenic, barium, cadmium, calcium, cobalt, copper, chromium, iron, lead, magnesium, manganese, molybdenum, nickel, rubidium, selenium, strontium, titanium, tin, vanadium, and zinc) were measured in both cohorts. The samples with concentrations less than the limit of detection were given a value equal to the limit of detection divided by √2 as concentrations. More than 20% of samples in FAMHES did not reach the limit of detection for antimony levels. Therefore, in the subsequent analysis, we included the remaining 21 metals, excluding antimony.

### Genotyping, imputation, and quality control

In our study, a total of 2020 subjects were chosen from FAMHES and genotyped using the Illumina Omini one platform^93^. We excluded individuals with (1) individuals call rate <0.95, (2) genotypic and phenotypic sex mismatch, (3) heterozygosity rate deviating more than three standard deviations from the mean, (4) kinship coefficient > 0.0884, (5) missingness of phenotypes or covariates. We also excluded SNPs with (1) call rate <0.95, (2) MAF < 0.05, (3) Hardy–Weinberg equilibrium *P*-value (*P*_HWE_) < 1.0 × 10^−6^. Then we used liftOverPlink (https://github.com/sritchie73/liftOverPlink) to convert genome position from hg18 to hg19. Snpflip (https://github.com/biocore-ntnu/snpflip) was used to fix the problem with reverse and ambiguous strand SNPs. Before phasing and imputation, we redid the same quality control procedure mentioned above. Eagle v2.4.1 (https://alkesgroup.broadinstitute.org/Eagle/) and Minimac4 (https://github.com/statgen/Minimac4) were used to phase and impute genotyped data of autosomal chromosomes, respectively. In the procedure of phasing and imputation, we chose 1000 G Phase3 v5 EAS (GRCh37/hg19) as a reference panel. After phasing and imputation, SNPs with (1) MAF < 0.01, (2) minor allele count < 20, (3) *P*_HWE_ < 1.0 × 10^−6^, (4) imputation quality (Rsq) < 0.7 were excluded. A total of 805 subjects were included from MEWHC and genotyped using Infinium Asian Screening Array-24 + v1.0 Kit (ASA). Because of the lack of X chromosome genotype data, we did not compare whether the genotypic and the phenotypic sex match. The remaining procedure of quality control was the same as what was done in FAMHES. Finally, before conducting GWAS, we removed the outliers whose metal concentrations were more than 4.5 inter-quartile ranges away from the median of each metal concentration. The rank-based inverse normal transformation was used to transform each metal concentration to approximate normality^94–96^.

### GWAS and meta-analysis

For each metal, we conducted a GWAS respectively in both cohorts using a generalized linear model adjusting age, sex, body mass index, ethnicity, first ten principal components, alcoholic drinking, and smoking status. PLINK 2.0 (www.cog-genomics.org/plink/2.0/) was selected to perform the GWAS under assumptions of dosage additive effects^97^. *P* = 5.0 × 10^−8^ was set as the genome-wide significance threshold, and *P* = 1.0 × 10^−5^ was selected as the genome-wide suggestive threshold. Stepwise conditional analysis was conducted to find the additional independent significant signal. For replication, we planned to perform meta-analyses using GWAS summary statistics of the same metal from both FAMHES and MEWHC. However, considering the difference between serum and plasma metal concentrations, we conducted the non-parametric test on the concentrations of each metal between the two cohorts via IBM SPSS Statistics 25 (https://www.ibm.com/support/pages/downloading-ibm-spss-statistics-25). Finally, since there was no statistically significant difference in copper concentrations and manganese concentrations between the two cohorts, we respectively conducted GWAS meta-analysis on the copper and manganese levels. Because of the significant statistical difference between the concentrations of other same metals in the two cohorts, the meta-analysis was not performed in the remaining metals. The Bonferroni-corrected threshold of < 2.38 × 10^−9^ (5.0 × 10^−8^/(21 metals)) in the context of meta-analysis was considered. Since we did not find any SNP significantly associated with manganese levels in the results of GWAS meta-analysis on manganese levels, and find two SNPs significantly associated with copper levels in the results of GWAS meta-analysis on copper levels, we only conducted the subsequent analysis of blood copper.

### Post-GWAS analysis

FUMA is an internet-based platform developed to annotate, prioritize, visualize, and interpret GWAS results^98,99^. We conducted annotation, functional mapping, gene-based analysis, gene-set analysis for the results of GWAS meta-analysis of SCLs via SNP2GENE function module of FUMA^100–102^, and then we used both mapped genes and the significant gene from gene-based analysis to construct gene expression heatmap via GENE2FUNC function module of FUMA to explore their expression states in various tissues^103,104^.

### MR

To investigate the causal relationships between SCLs and 213 deep phenotypes (152 diseases, 38 biomarkers, and 23 medication usages) from BioBank Japan in the East Asian population^20^, we performed bidirectional MR analysis. Detailed information about the 213 deep phenotypes is shown in Supplementary Data 3, and their GWAS summary statistics can be downloaded from the website (https://pheweb.jp/downloads).

In the first round two-sample MR analyses, we set SCLs as exposure and 213 deep phenotypes as outcomes. To identify the genetic IVs, we firstly selected genetic variants which were associated (*P* < 1.0 × 10^−5^) with SCLs from GWAS meta-analysis as candidate IVs^105^. Secondly, we filtered out those SNPs in strong LD since strong linkage disequilibrium will bias the subsequent analysis. In our study, we conducted LD clumping process (window size = 10,000 kb, *r*^2^ < 0.001) using 1000G Phase3 v5 EAS (GRCh37/hg19) as a reference panel, and SNPs with *r*^2^ < 0.001, MAF > 0.01 were retained. Thirdly, we evaluated the heterogeneity of each candidate IV using *I*^2^ and *P*-value for Cochran’s Q statistic. F-statistic was considered to avoid weak IV bias. In this step, we included SNPs with *I*^2^ < 50, *P*-value for Cochran’s *Q* statistic > 0.05, and *F*-statistic >10^106^. Fourthly, in the harmonizing process of two-sample MR analyses, both ambiguous and palindrome SNPs would be corrected or directly excluded. Since there was no need, we did not use any proxy SNPs. Finally, a total of 11 SNPs were included as IVs in the first round two-sample MR analyses. The total variance explained by the selected 11 SNPs was calculated using the following equation (Eq. 1):

1 $${{R}}_{{{{{{\rm{total}}}}}}}^{2}={\sum }_{i=1}^{n}2{f}_{n}(1-{f}_{n}){\beta }_{n}^{2},$$

where ${{R}}_{{{{{{\rm{total}}}}}}}^{2}$ referred to the total variance explained by the selected 11 SNPs, *f*_*n*_ referred to the effect allele frequency, *n* referred to the number of the selected SNPs (*n* = 11), and *β*_*n*_ referred to the additive effect^94^. All the 11 SNPs totally explained 11.09% of the transformed SCLs variance (Supplementary Table 12).

To further research whether the 213 deep phenotypes from BioBank Japan have causal effects on SCLs, we conducted the second round two-sample MR analyses, which is an inverse MR analysis comparing with the first round. We selected genetic variants which were associated (*P* < 1.0 × 10^−8^) with each phenotype from the GWAS summary statistics in BioBank Japan as candidate IVs. The rest of the process of selecting genetic IVs is the same as that done in the first round.

To comprehensively determine the causal effects of BCLs on these 213 deep phenotypes in humans, we further used the GWAS meta-analyses summary statistics of UK Biobank and FinnGen to perform the third round of two-sample MR analyses. The GWAS meta-analysis summary statistics could be downloaded at the website (https://pheweb.jp/downloads). Because of lacking serum/plasma copper levels GWAS summary statistics for European populations, we finally used the SNPs which were independently (*r*^2^ < 0.05) associated (*P* < 1.0 × 10^−5^) with ECLs as IVs^6^. Except that the reference panel used in the LD clumping process is 1000G Phase3 v5 EUR (GRCh37/hg19), the rest process of selecting genetic IVs is the same as that done in the first round. The details of the 12 SNPs, which were finally included as genetic IVs, are shown in Supplementary Table 13.

Because of the lack of GWAS summary statistics for the 38 biomarkers, we only conducted the two-sample MR analyses between ECLs and 174 deep phenotypes (151 diseases and 23 medication usage) in the end. More information about the 174 deep phenotypes is shown in Supplementary Data 4.

**Fig. 6:**
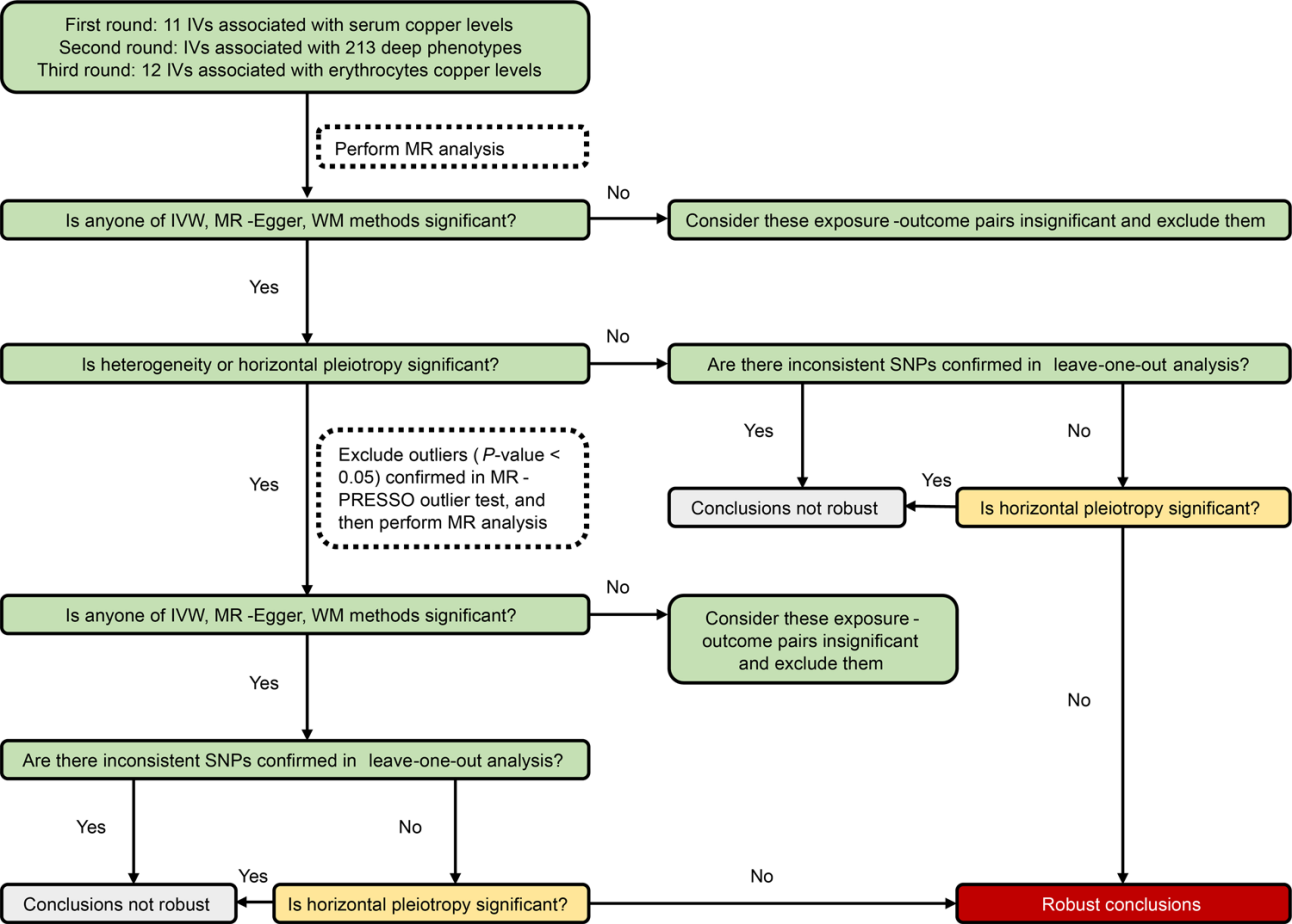
Flow chart of the two-sample Mendelian randomization analysis in East Asian and European populations. IVs instrumental variables, MR Mendelian randomization, IVW inverse-variance weighted, WM weighted median, MR-PRESSO Mendelian randomization pleiotropy residual sum and outlier.

All three rounds of two-sample MR analyses followed the flow chart (Fig. 6). Firstly, we used IVW, MR-Egger, and WM methods of the TwoSampleMR package to identify exposure-outcome pairs that might have causal relationships^107–110^. Significant (*P* < 0.05) difference between *y*-intercept of MR-Egger and 0 represents that there is significant horizontal pleiotropy. *P*-value of Cochran’s Q statistics of IVW and MR-Egger < 0.05 represents that there is significant heterogeneity. If there was significant horizontal pleiotropy or heterogeneity, the MR-PRESSO outlier test would be used to detect the outlier, which usually leads to horizontal pleiotropy^111^. The NbDistribution (number of elements to simulate to form the null distribution to compute empirical *P*-values) in the MR-PRESSO test was set to 20,000. After removing the outliers, we re-performed two-sample MR analyses with IVW, MR-Egger, and WM methods. Based on the results of the leave-one-out sensitivity analysis, horizontal pleiotropy tested via MR-Egger, and heterogeneity tested via IVW and MR-Egger, we divide the results into the section where robust conclusions can be drawn and the section where robust conclusions cannot be drawn. Then we performed MR-RAPS to re-estimate causality in exposure-outcome pairs of robust conclusions section to eliminate the estimation bias of weak IVs^112^. In addition, we also considered the corresponding Bonferroni-corrected threshold < 0.00023 (0.05/213 outcomes) and the suggestively significant threshold (0.00023 ≤ *P* ≤ 0.05) in MR analysis. Finally, we calculated the statistical power of IVW, MR-Egger, MR-RAPS, and WM methods using the methods which are based on the non-centrality parameter^113,114^. Because of the lacking variance of the exposure variable and outcome variable, we didn’t calculate the statistical power for quantitative trait outcomes. All two-sample MR analyses were performed in R version 4.0.5 using the TwoSampleMR package^109^. A two-sided *P* value < 0.05 was used to determine statistical significance.

### Reporting summary

Further information on research design is available in the Nature Research Reporting Summary linked to this article.

## Supplementary information

Supplementary Information

Description of Additional Supplementary Files

Supplementary Data 1

Supplementary Data 2

Supplementary Data 3

Supplementary Data 4

Reporting Summary

## Data Availability

The GWAS summary statistics of 213 deep phenotypes can be downloaded from the website (https://pheweb.jp/downloads). The GWAS summary statistics of serum/plasma metal levels have been provided to the NHGRI-EBI GWAS Catalog and the study accession numbers are GCST90100517, GCST90100518, GCST90100519, GCST90100520, GCST90100521, GCST90100522, GCST90100523, GCST90100524, GCST90100525, GCST90100526, GCST90100527, GCST90100528, GCST90100529, GCST90100530, GCST90100531, GCST90100532, GCST90100533, GCST90100534, GCST90100535, GCST90100536, GCST90100537, GCST90100538, GCST90100539, GCST90100540, GCST90100541, GCST90100542, GCST90100543, GCST90100544, GCST90100545, GCST90100546, GCST90100547, GCST90100548, GCST90100549, GCST90100550, GCST90100551, GCST90100552, GCST90100553, GCST90100554, GCST90100555, GCST90100556, GCST90100557, GCST90100558. The source data for graphs and charts can be downloaded from Figshare (https://figshare.com) through the 10.6084/m9.figshare.17696942. The datasets generated and analyzed during the current study are available in the Genome variation Map (GVM) of National Genomics Data Center (NGDC) (Accession Number: GVM000052). Please contact the corresponding author for more information if necessary.

## Code availability

The liftOverPlink (https://github.com/sritchie73/liftOverPlink); Snpflip (https://github.com/biocore-ntnu/snpflip); Eagle v2.4.1 (https://alkesgroup.broadinstitute.org/Eagle/); Minimac4 (https://github.com/statgen/Minimac4); PLINK 2.0 (www.cog-genomics.org/plink/2.0/); IBM SPSS Statistics 25 (https://www.ibm.com/support/pages/downloading-ibm-spss-statistics-25); FUMA (https://fuma.ctglab.nl); R (https://www.r-project.org).
