# Supplementary material for: Genome-wide association and Mendelian randomization study of blood copper levels and 213 deep phenotypes in humans
Source: Commun Biol. 2022 May 2;5:405. doi: 10.1038/s42003-022-03351-7 (PMC9061855; doi:10.1038/s42003-022-03351-7)
Supplement: Supplementary file 1 — Supplementary Information [file 42003_2022_3351_MOESM1_ESM.pdf]

Genome-wide association and Mendelian randomization study of blood copper levels and 213 deep phenotypes in humans

Wenjun Yang [1, 2, 3, #], Longman Li [1, 3, #], Xiuming Feng [1, 3, #], Hong Cheng [1,3], Xiaoting Ge [1, 3], Yu Bao [1, 3], Lulu Huang [1, 4], Fei Wang [3, 5], Chaoqun Liu [6], Xing Chen [7], Zengnan Mo [1, 8], Xiaobo Yang [1, 3, 9, *]

[1] Center for Genomic and Personalized Medicine, Guangxi key Laboratory for Genomic and Personalized Medicine, Guangxi Collaborative Innovation Center for Genomic and Personalized Medicine, Guangxi Medical University, Nanning 530021, Guangxi, China

[2] Collaborative Innovation Centre of Regenerative Medicine and Medical BioResource Development and Application, Guangxi Medical University, Nanning, Guangxi 530021, China

[3] Department of Occupational Health and Environmental Health, School of Public Health, Guangxi Medical University, Nanning, Guangxi, China

[4] Department of radiotherapy, First Affiliated Hospital of Guangxi Medical University, Nanning, China

[5] Guangxi key Laboratory for Thyroid Tumor Precision Prevention and Treatment, Liuzhou, Guangxi, China

[6] Department of Nutrition and Food Hygiene, School of Public Health, Guangxi Medical University, Nanning, Guangxi, China

[7] School of Public Health, Guangxi Medical University, Nanning, Guangxi, China

[8] Department of Urology, Institute of Urology and Nephrology, First Affiliated Hospital of Guangxi Medical University, Nanning, Guangxi, China

[9] Department of Public Health, School of Medicine, Guangxi University of Science and Technology, Liuzhou, Guangxi, China

[*] Corresponding author: Xiaobo Yang, Department of Occupational Health and Environmental Health, School of Public Health, Guangxi Medical University, Nanning, 530021, Guangxi, China. Email: yangx@gxmu.edu.cn. Telephone number: +8618677146179.

[#] These authors contributed equally to this article.

Supplementary Table 1. Top SNPs (P < 5E-8) associated with 21 serum/plasma metal levels in FAMHES and MEWHC.

Cohort	Metal	CHR	POS (hg19)	SNP	Gene	EA	NEA	EAF	N	BETA	SE	*P*
FAMHES	Serum copper levels	12	112337924	rs78069066	*ADAM1A*	A	G	0.263	1798	-0.240	0.040	3.58E-09
	Serum copper levels	19	14936237	rs10424895	*OR7A5* (-0.901kb)	A	G	0.306	1798	0.198	0.036	4.42E-08
MEWHC	Plasma iron levels	22	37462926	rs2235321	*TMPRSS6*	A	G	0.375	687	0.318	0.053	3.20E-09

Abbreviations: CHR, chromosome; POS, position; SNP, single-nucleotide polymorphism; EA, effect allele; NEA, non-effect allele; EAF, effect allele frequency; N, the sample size of the genome-wide association studies or meta-analysis; FAMHES, the Fangchenggang Area Male Health and Examination Survey; MEWHC, the manganese-exposed workers healthy cohort.

Supplementary Table 2. Significant independent SNPs (r^2 < 0.6) identified from GWAS meta-analysis of serum copper levels.

Genomic Loci	uniqID	SNP	CHR	POS (hg19)	*P*	nSNPs	nGWASSNPs
1	3:148940672:C:T	rs35691438	3	148940672	1.63E-09	31	16
2	12:111705893:A:G	rs3858704	12	111705893	2.39E-09	5	5
2	12:112140669:A:G	rs11066008	12	112140669	2.25E-08	6	1
2	12:112241766:A:G	rs671	12	112241766	2.21E-10	19	10

Abbreviations: SNP, single-nucleotide polymorphism; CHR, chromosome; POS, position.
Note: Genomic loci: the index of genomic risk loci;
nSNPs: the number of SNPs in LD with the independent significant SNPs (r^2<0.6), including non-GWAS-tagged SNPs which are extracted from 1000 Genomes Project Phase 3 (EAS);
nGWASSNPs: the number of GWAS-tagged SNPs in LD with the independent significant SNPs (r^2<0.6).

Supplementary Table 3. Results of gene-based analysis based on GWAS meta-analysis of serum copper levels.

GENE	CHR	Start (hg19)	Stop (hg19)	NSNPS	NPARAM	N	ZSTAT	*P*	SYMBOL
ENSG00000047457	3	148880197	148939842	67	9	2483	4.6176	1.94E-06	CP
ENSG00000089169	12	113008184	113336686	383	29	2483	4.5647	2.50E-06	RPH3A
ENSG00000127515	19	14951760	14952689	5	2	2483	4.4465	4.36E-06	OR7A10
ENSG00000127329	12	70910630	71031220	249	31	2483	4.0596	2.46E-05	PTPRB
ENSG00000188269	19	14903302	14946188	109	16	2483	3.7916	7.48E-05	OR7A5
ENSG00000157093	3	42438570	42452092	14	3	2483	3.5886	1.66E-04	LYZL4
ENSG00000188778	8	37820516	37824483	4	1	2483	3.5798	1.72E-04	ADRB3
ENSG00000175643	16	11343476	11445619	303	8	2483	3.5172	2.18E-04	RMI2
ENSG00000159216	21	36160098	37376965	1751	98	2483	3.516	2.19E-04	RUNX1
ENSG00000177576	18	47008028	47013622	9	3	2483	3.4965	2.36E-04	C18orf32
ENSG00000139737	13	78272023	78338377	93	8	2483	3.4944	2.38E-04	SLAIN1
ENSG00000189366	3	125648118	125655882	32	2	2483	3.4608	2.69E-04	ALG1L
ENSG00000111245	12	111348623	111358526	31	1	2483	3.451	2.79E-04	MYL2
ENSG00000175646	16	11374693	11375207	1	1	2483	3.4501	2.80E-04	PRM1
ENSG00000132274	11	5710919	5758319	140	16	2483	3.3988	3.38E-04	TRIM22
ENSG00000166444	11	8714898	8932498	341	18	2483	3.3957	3.42E-04	ST5
ENSG00000178279	16	11361605	11363390	3	1	2483	3.3826	3.59E-04	TNP2
ENSG00000114547	3	125687987	125702297	19	4	2483	3.3749	3.69E-04	ROPN1B
ENSG00000122304	16	11369496	11370337	2	1	2483	3.3283	4.37E-04	PRM2
ENSG00000182584	20	32254304	32256331	2	1	2483	3.3202	4.50E-04	ACTL10
ENSG00000089234	12	112079950	112123790	24	3	2483	3.3152	4.58E-04	BRAP

ENSG00000215472	18	47008051	47017956	23	4	2483	3.2872	5.06E-04	RPL17-C18orf32
ENSG00000101391	20	31946645	31989367	22	4	2483	3.2694	5.39E-04	CDK5RAP1
ENSG00000168303	7	40165622	40174258	4	1	2483	3.2507	5.76E-04	MPLKIP
ENSG00000184588	1	66258197	66840259	1094	55	2483	3.211	6.61E-04	PDE4B
ENSG00000230062	6	46714654	46727243	11	3	2483	3.2074	6.70E-04	ANKRD66
ENSG00000111275	12	112204691	112247782	46	5	2483	3.2021	6.82E-04	ALDH2
ENSG00000173093	12	111284573	111345339	111	6	2483	3.1938	7.02E-04	CCDC63
ENSG00000127980	7	92116334	92157845	12	2	2483	3.1218	8.99E-04	PEX1
ENSG00000115339	2	166604101	166651192	59	7	2483	3.1139	9.23E-04	GALNT3

Note: NSNPS: the number of SNPs annotated to that gene that were found in the data and were not excluded based on internal SNP QC; NPARAM: the number of relevant parameters used in the model; N: the sample size used when analyzing that gene.

Supplementary Table 4. Results of gene-set analysis based on GWAS meta-analysis of serum copper levels.

GENE SET	TYPE	NGENES	BETA	BETA_STD	SE	*P*
GO_bp:go_regulation_of_protein_oligomerization	SET	40	0.574	0.028	0.134	8.79E-06
GO_bp:go_negative_regulation_of_antigen_processing_and_presentation	SET	8	1.168	0.025	0.279	1.38E-05
Curated_gene_sets:reactome_microautophagy	SET	25	0.640	0.024	0.155	1.76E-05
Curated_gene_sets:kokkinakis_methionine_deprivation_96hr_up	SET	112	0.335	0.027	0.086	5.06E-05
GO_mf:go_small_ribosomal_subunit_rrna_binding	SET	7	1.309	0.026	0.339	5.61E-05
GO_bp:go_regulation_of_tubulin_deacetylation	SET	8	1.078	0.023	0.283	6.83E-05
GO_bp:go_positive_regulation_of_viral_process	SET	96	0.338	0.025	0.090	8.53E-05
GO_bp:go_single_fertilization	SET	125	0.279	0.024	0.075	1.01E-04
GO_bp:go_tubulin_deacetylation	SET	9	1.008	0.023	0.272	1.04E-04
Curated_gene_sets:marks_acetylated_non_histone_proteins	SET	11	1.072	0.027	0.299	1.67E-04
Curated_gene_sets:kokkinakis_methionine_deprivation_48hr_up	SET	122	0.293	0.024	0.082	1.71E-04
Curated_gene_sets:wei_mycn_targets_with_e_box	SET	714	0.117	0.023	0.033	1.71E-04
GO_mf:go_calcium_ion_binding	SET	626	0.135	0.025	0.038	1.79E-04
Curated_gene_sets:chiba_response_to_tsa_dn	SET	20	0.720	0.024	0.208	2.61E-04
Curated_gene_sets:kommagani_tp63_gamma_targets	SET	9	0.997	0.023	0.287	2.61E-04
Curated_gene_sets:schavolt_targets_of_tp53_and_tp63	SET	12	0.793	0.021	0.232	3.20E-04
Curated_gene_sets:reactome_regulation_of_gene_expression_in_late_stage_branching_ morphogenesis_pancreatic_bud_precursor_cells	SET	15	0.895	0.026	0.265	3.63E-04
Curated_gene_sets:biocarta_p53_pathway	SET	15	0.776	0.023	0.231	3.87E-04
Curated_gene_sets:pid_hes_hey_pathway	SET	42	0.470	0.023	0.141	4.19E-04
GO_cc:go_cytolytic_granule	SET	5	1.242	0.021	0.372	4.25E-04

GO_cc:go_atpase_complex	SET	87	0.313	0.022	0.094	4.51E-04
GO_bp:go_gas_homeostasis	SET	9	0.889	0.020	0.268	4.53E-04
GO_bp:go_negative_regulation_of_protein_oligomerization	SET	16	0.724	0.022	0.218	4.59E-04
GO_bp:go_positive_regulation_of_viral_release_from_host_cell	SET	14	0.728	0.021	0.220	4.75E-04
Curated_gene_sets:smid_breast_cancer_relapse_in_pleura_up	SET	3	1.940	0.026	0.588	4.82E-04
GO_bp:go_regulation_of_antigen_processing_and_presentation	SET	14	0.713	0.020	0.220	5.90E-04
Curated_gene_sets:howlin_pubertal_mammary_gland	SET	58	0.396	0.023	0.122	6.01E-04
GO_cc:go_sm_like_protein_family_complex	SET	65	0.337	0.021	0.104	6.10E-04
GO_bp:go_postsynaptic_modulation_of_chemical_synaptic_transmission	SET	16	0.624	0.019	0.193	6.23E-04
GO_cc:go_prespliceosome	SET	15	0.749	0.022	0.234	6.82E-04

Note: TYPE: denotes the type of variable, either SET or COVAR for normal gene sets and gene covariates provided in the input files; or INTER-SS or INTER-SC, for internally created interaction terms (set by set and set by covariate, respectively);
NGENES: the number of genes in the data that are in the set (for gene sets and set by covariate interactions), that are in the interaction set (for set by set interactions), or for which non-missing values were available (for gene covariates).

Supplementary Table 5. 21 exposure-outcome pairs that may have causal relationships from the first round two-sample Mendelian randomization analyses.

Exposure	Outcome	SNPs	IVW				MR-Egger						WM	
			OR (95% CI)	*P* value	Cochran Q statistics (df)	*P* value	OR (95% CI)	*P* value	Intercept (Se)	*P* value	Cochran Q statistics (df)	*P* value	OR (95% CI)	*P* value
SCLs	Beta blocking agents	11	0.918 (0.844 0.999)	0.047	30.823 (10)	0.0006	0.868 (0.612 1.231)	0.447	0.011 (0.034)	0.752	30.465 (9)	0.0003	0.962 (0.896 1.034)	0.293
	Drugs affecting bone structure and mineralization	11	0.887 (0.813 0.967)	0.007	11.275 (10)	0.336	0.700 (0.509 0.963)	0.056	0.046 (0.031)	0.168	9.021 (9)	0.435	0.867 (0.774 0.970)	0.013
	Immunosuppressants	11	0.865 (0.750 0.997)	0.046	17.212 (10)	0.070	0.926 (0.512 1.673)	0.804	-0.013 (0.057)	0.820	17.109 (9)	0.047	0.897 (0.762 1.056)	0.191
	Allergic conjunctivitis	11	1.021 (0.884 1.178)	0.781	19.976 (10)	0.029	1.773 (1.111 2.830)	0.040	-0.108 (0.045)	0.040	12.199 (9)	0.202	0.999 (0.855 1.167)	0.991
	Brain tumor	11	1.240 (0.825 1.865)	0.300	17.485 (10)	0.064	5.422 (1.373 21.412)	0.039	-0.288 (0.132)	0.058	11.459 (9)	0.246	1.383 (0.899 2.128)	0.140
	Breast cancer	11	0.915 (0.842 0.995)	0.038	6.864 (10)	0.738	1.081 (0.777 1.502)	0.656	-0.032 (0.032)	0.334	5.824 (9)	0.757	0.922 (0.822 1.034)	0.165
	Chronic gastritis	11	1.284 (1.022 1.613)	0.032	9.013 (10)	0.531	1.758 (0.717 4.307)	0.249	-0.061 (0.086)	0.496	8.510 (9)	0.484	1.339 (0.991 1.811)	0.058
	Compression fracture	11	1.153 (1.024 1.298)	0.018	5.824 (10)	0.830	1.015 (0.636 1.620)	0.950	0.025 (0.045)	0.594	5.520 (9)	0.787	1.149 (0.983 1.342)	0.080
	Esophageal varix	11	1.998 (1.317 3.032)	0.001	11.948 (10)	0.289	6.479 (1.394 30.108)	0.041	-0.230 (0.148)	0.155	9.427 (9)	0.399	1.773 (0.988 3.182)	0.055
	Food allergy	11	0.803 (0.692 0.932)	0.004	20.914 (10)	0.022	0.776 (0.418 1.443)	0.444	0.007 (0.060)	0.913	20.885 (9)	0.013	0.873 (0.743 1.026)	0.100
	Glaucoma	11	1.107 (1.031 1.190)	0.005	8.752 (10)	0.556	1.268 (0.959 1.675)	0.130	-0.027 (0.027)	0.351	7.784 (9)	0.556	1.110 (1.009 1.221)	0.032
	Hepatic cancer	11	1.226 (1.005 1.496)	0.045	21.321 (10)	0.019	1.277 (0.554 2.945)	0.580	-0.008 (0.080)	0.924	21.298 (9)	0.011	1.098 (0.902 1.336)	0.353
	Malignant lymphoma	11	0.708 (0.503 0.995)	0.047	9.289 (10)	0.505	0.491 (0.128 1.890)	0.328	0.071 (0.130)	0.597	8.988 (9)	0.438	0.650 (0.412 1.027)	0.065
	Periodontal disease	11	1.062 (0.994 1.135)	0.076	10.209 (10)	0.422	1.328 (1.025 1.722)	0.061	-0.044 (0.025)	0.115	7.163 (9)	0.620	1.096 (1.002 1.199)	0.046
	Rheumatoid arthritis	11	0.881 (0.799 0.971)	0.010	12.094 (10)	0.279	1.030 (0.698 1.521)	0.884	-0.031 (0.038)	0.435	11.261 (9)	0.258	0.873 (0.767 0.993)	0.039
	Sarcoidosis	11	0.894 (0.540 1.480)	0.663	14.385 (10)	0.156	0.072 (0.014 0.375)	0.012	0.493 (0.160)	0.013	4.830 (9)	0.849	0.676 (0.365 1.253)	0.214
	Sleep apnea syndrome	11	1.615 (1.212 2.153)	0.001	2.815 (10)	0.985	1.042 (0.335 3.241)	0.945	0.086 (0.109)	0.454	2.202 (9)	0.988	1.567 (1.070 2.294)	0.021
	Systemic lupus erythematosus	11	2.058 (1.422 2.980)	0.0001	11.266 (10)	0.337	5.412 (1.344 21.788)	0.041	-0.189 (0.134)	0.193	9.235 (9)	0.416	1.722 (1.072 2.766)	0.025
	Tonsillitis	11	1.395 (1.027 1.895)	0.033	9.567 (10)	0.479	1.879 (0.545 6.474)	0.344	-0.058 (0.119)	0.638	9.321 (9)	0.408	1.485 (0.971 2.270)	0.068
	Urticaria	11	0.929 (0.864 0.999)	0.047	12.311 (10)	0.265	0.949 (0.702 1.283)	0.740	-0.004 (0.029)	0.894	12.285 (9)	0.198	0.923 (0.835 1.021)	0.119
	White blood cell count (WBC)*	11	-0.037 (-0.079 0.004)	0.079	77.932 (10)	1.27E-12	-0.043 (-0.214 0.128)	0.635	0.001 (0.017)	0.947	77.892 (9)	4.23E-13	-0.025 (-0.050 -0.001)	0.044

Abbreviations: SCLs, serum copper levels; MR, Mendelian randomization; SNPs, number of single-nucleotide polymorphism used as instrumental variables; IVW, inverse-variance weighted; WM, weighted median.
Note: * OR and 95% confidence interval are replaced by β and 95% confidence interval.

Supplementary Table 6. Results where robust conclusions cannot be drawn of the first round two-sample Mendelian randomization analyses.

Exposure	Outcome	SNPs	IVW				MR-Egger						WM	
			OR (95% CI)	*P* value	Cochran Q statistics (df)	*P* value	OR (95% CI)	*P* value	Intercept (Se)	*P* value	Cochran Q statistics (df)	*P* value	OR (95% CI)	*P* value
SCLs	Immunosuppressants	11	0.865 (0.750 0.997)	0.046	17.212 (10)	0.070	0.926 (0.512 1.673)	0.804	-0.013 (0.057)	0.820	17.109 (9)	0.047	0.897 (0.762 1.056)	0.191
	Allergic conjunctivitis	11	1.021 (0.884 1.178)	0.781	19.976 (10)	0.029	1.773 (1.111 2.830)	0.040	-0.108 (0.045)	0.040	12.199 (9)	0.202	0.999 (0.855 1.167)	0.991
	Brain tumor	11	1.240 (0.825 1.865)	0.300	17.485 (10)	0.064	5.422 (1.373 21.412)	0.039	-0.288 (0.132)	0.058	11.459 (9)	0.246	1.383 (0.899 2.128)	0.140
	Breast cancer	11	0.915 (0.842 0.995)	0.038	6.864 (10)	0.738	1.081 (0.777 1.502)	0.656	-0.032 (0.032)	0.334	5.824 (9)	0.757	0.922 (0.822 1.034)	0.165
	Chronic gastritis	11	1.284 (1.022 1.613)	0.032	9.013 (10)	0.531	1.758 (0.717 4.307)	0.249	-0.061 (0.086)	0.496	8.510 (9)	0.484	1.339 (0.991 1.811)	0.058
	Compression fracture	11	1.153 (1.024 1.298)	0.018	5.824 (10)	0.83	1.015 (0.636 1.620)	0.950	0.025 (0.045)	0.594	5.520 (9)	0.787	1.149 (0.983 1.342)	0.080
	Food allergy	10	0.863 (0.762 0.977)	0.020	11.163 (9)	0.265	0.886 (0.543 1.447)	0.642	0.007 (0.060)	0.913	11.146 (8)	0.194	0.915 (0.779 1.075)	0.280
	Malignant lymphoma	11	0.708 (0.503 0.995)	0.047	9.289 (10)	0.505	0.491 (0.128 1.890)	0.328	0.071 (0.130)	0.597	8.988 (9)	0.438	0.650 (0.412 1.027)	0.065
	Periodontal disease	11	1.062 (0.994 1.135)	0.076	10.209 (10)	0.422	1.328 (1.025 1.722)	0.061	-0.044 (0.025)	0.115	7.163 (9)	0.620	1.096 (1.002 1.199)	0.046
	Rheumatoid arthritis	11	0.881 (0.799 0.971)	0.010	12.094 (10)	0.279	1.030 (0.698 1.521)	0.884	-0.031 (0.038)	0.435	11.261 (9)	0.258	0.873 (0.767 0.993)	0.039
	Sarcoidosis	11	0.894 (0.540 1.480)	0.663	14.385 (10)	0.156	0.072 (0.014 0.375)	0.012	0.493 (0.160)	0.013	4.830 (9)	0.849	0.676 (0.373 1.224)	0.196
	Tonsillitis	11	1.395 (1.027 1.895)	0.033	9.567 (10)	0.479	1.879 (0.545 6.474)	0.344	-0.058 (0.119)	0.638	9.321 (9)	0.408	1.485 (0.971 2.270)	0.068
	Urticaria	11	0.929 (0.864 0.999)	0.047	12.311 (10)	0.265	0.949 (0.702 1.283)	0.740	-0.004 (0.029)	0.894	12.285 (9)	0.198	0.923 (0.835 1.021)	0.119

Abbreviations: SCLs, serum copper levels; MR, Mendelian randomization; SNPs, number of single-nucleotide polymorphism used as instrumental variables; IVW, inverse-variance weighted; WM, weighted median.
Note: * OR and 95% confidence interval are replaced by β and 95% confidence interval.

Supplementary Table 7. 16 exposure-outcome pairs that may have causal relationships from the second round two-sample Mendelian randomization (MR) analyses.

Exposure	Outcome	SNPs	IVW				MR-Egger						WM	
			β (95% CI)	*P* value	Cochran Q statistics (df)	*P* value	β (95% CI)	*P* value	Intercept (Se)	*P* value	Cochran Q statistics (df)	*P* value	β (95% CI)	*P* value
Calcium channel blockers		16	0.294 (-0.092 0.679)	0.136	38.953 (15)	0.001	2.179 (0.887 3.470)	0.005	-0.137 (0.046)	0.011	24.032 (14)	0.045	0.073 (-0.273 0.418)	0.680
Angina pectoris		17	-0.118 (-0.384 0.148)	0.384	47.493 (16)	5.70E-05	-1.002 (-1.918 -0.085)	0.049	0.096 (0.049)	0.069	37.810 (15)	0.001	0.035 (-0.188 0.258)	0.758
Ischemic stroke		3	1.099 (-0.0004 2.199)	0.050	14.449 (2)	0.001	4.458 (2.221 6.695)	0.160	-0.285 (0.095)	0.204	1.431 (1)	0.232	0.728 (0.060 1.395)	0.033
Myocardial infarction		30	-0.099 (-0.234 0.036)	0.149	60.283 (29)	0.001	-0.582 (-0.832 -0.333)	8.77E-05	0.072 (0.017)	0.0002	36.925 (28)	0.121	0.025 (-0.132 0.183)	0.752
Albumin (Alb)		20	0.197 (-0.261 0.656)	0.399	25.633 (19)	0.141	-1.014 (-1.895 -0.132)	0.037	0.054 (0.018)	0.007	16.564 (18)	0.553	-0.155 (-0.720 0.411)	0.592
Alanine aminotransferase (ALT)		19	0.619 (0.057 1.181)	0.031	21.612 (18)	0.250	-0.673 (-2.698 1.351)	0.523	0.039 (0.030)	0.211	19.657 (17)	0.292	0.624 (-0.151 1.400)	0.114
Body mass index (BMI)		57	0.363 (0.038 0.688)	0.029	49.009 (56)	0.735	0.612 (-0.362 1.587)	0.223	-0.007 (0.013)	0.597	48.726 (55)	0.712	0.320 (-0.193 0.832)	0.221
Diastolic blood pressure (DBP)		16	1.080 (0.277 1.883)	0.008	32.259 (15)	0.006	3.329 (1.549 5.110)	0.003	-0.078 (0.029)	0.018	21.343 (14)	0.093	0.638 (-0.243 1.519)	0.156
Gamma-glutamyl transpeptidase (GGT)	SCLs	35	0.517 (0.230 0.804)	0.0004	30.822 (34)	0.624	0.226 (-0.316 0.767)	0.420	0.014 (0.011)	0.222	29.273 (33)	0.653	0.631 (0.188 1.074)	0.005
Mean arterial pressure (MAP)		25	0.611 (-0.049 1.272)	0.070	52.122 (24)	0.001	3.493 (2.101 4.884)	5.70E-05	-0.095 (0.022)	0.0002	28.605 (23)	0.194	0.526 (-0.215 1.267)	0.164
Mean corpuscular hemoglobin concentration (MCHC)		35	0.392 (0.003 0.780)	0.048	54.755 (34)	0.014	0.693 (-0.208 1.593)	0.141	-0.013 (0.018)	0.472	53.891 (33)	0.012	0.188 (-0.253 0.630)	0.404
Mean corpuscular volume (MCV)		88	0.150 (-0.028 0.328)	0.098	105.417 (87)	0.087	0.375 (0.034 0.715)	0.034	-0.012 (0.008)	0.134	102.685 (86)	0.106	0.111 (-0.159 0.381)	0.421
Neutrophil count (Neutro)		25	-0.297 (-0.646 0.051)	0.095	35.940 (24)	0.056	-1.052 (-1.902 -0.202)	0.024	0.041 (0.022)	0.071	31.108 (23)	0.120	-0.003 (-0.429 0.424)	0.991
Red blood cell count (RBC)		63	-0.341 (-0.622 -0.060)	0.017	79.342 (62)	0.068	-0.611 (-1.180 -0.042)	0.039	0.011 (0.010)	0.289	77.884 (61)	0.071	-0.140 (-0.572 0.292)	0.524
Sytolic blood pressure (SBP)		25	0.649 (-0.035 1.333)	0.063	53.524 (24)	0.0005	3.780 (2.128 5.431)	0.0002	-0.097 (0.025)	0.001	32.010 (23)	0.100	0.564 (-0.152 1.280)	0.123
White blood cell count (WBC)		53	-0.217 (-0.496 0.061)	0.127	49.045 (52)	0.591	-0.710 (-1.361 -0.059)	0.037	0.020 (0.012)	0.107	46.354 (51)	0.658	-0.231 (-0.683 0.221)	0.317

Abbreviations: SCLs, serum copper levels; MR, Mendelian randomization; SNPs, number of single-nucleotide polymorphism used as instrumental variables; IVW, inverse-variance weighted; WM, weighted median.

Supplementary Table 8. Summary of the instrumental variables of gamma-glutamyl transpeptidase levels.

Phenotype	CHR	POS (hg19)	SNP	EA	NEA	EAF	BETA	SE	*P*	N	R^2	F statistic
Gamma-glutamyl transpeptidase levels	1	16505320	rs1497406	A	G	0.194	-0.0586691	0.00439172	1.00E-40	133471	0.0011	143.98
	1	178529898	rs12753251	G	A	0.535	-0.0234463	0.00347058	1.40E-11	133471	0.0003	36.52
	1	200267292	rs10919884	G	A	0.559	-0.0238267	0.00350567	1.10E-11	133471	0.0003	37.37
	2	27730940	rs1260326	T	C	0.557	0.0556103	0.00349073	3.90E-57	133471	0.0015	204.05
	2	169859716	rs112779928	C	T	0.674	-0.0335484	0.00369213	1.00E-19	133471	0.0005	66.06
	2	233520254	rs13395911	A	T	0.671	-0.0335049	0.00379359	1.00E-18	133471	0.0005	66.22
	3	4909440	rs2053500	T	C	0.490	-0.0305943	0.00362246	3.00E-17	133471	0.0005	62.47
	4	3446883	rs3752442	A	G	0.569	0.0224854	0.00350216	1.40E-10	133471	0.0002	33.12
	5	31025013	rs1500175	T	C	0.872	0.0326854	0.00518314	2.90E-10	133471	0.0002	31.78
	5	52193237	rs870992	A	G	0.927	-0.054622	0.00666635	2.50E-16	133471	0.0004	53.92
	6	53939724	rs9349693	A	G	0.526	0.0310546	0.00347062	3.60E-19	133471	0.0005	64.21
	7	26022414	rs7780562	A	C	0.322	-0.032421	0.00385282	3.90E-17	133471	0.0005	61.32
	7	28219956	rs186735625	C	T	0.512	-0.0207021	0.00349528	3.20E-09	133471	0.0002	28.59
	7	73035857	rs7800944	T	C	0.895	0.0577882	0.00569637	3.50E-24	133471	0.0006	84.16
	7	97826232	rs1495525	G	A	0.482	0.0253066	0.0034767	3.40E-13	133471	0.0003	42.70
	8	126482077	rs2954021	A	G	0.452	0.0215459	0.00347496	5.60E-10	133471	0.0002	30.70
	10	79657705	rs7099526	C	G	0.772	-0.0475719	0.00415138	2.10E-30	133471	0.0008	106.36
	11	34695771	rs704735	C	T	0.672	-0.0212818	0.00372642	1.10E-08	133471	0.0002	26.64
	11	62199457	rs10897272	C	T	0.545	0.0303656	0.00348262	2.80E-18	133471	0.0005	61.06
	12	53274674	rs2682302	G	T	0.764	-0.0243793	0.00405426	1.80E-09	133471	0.0002	28.61

12	110172974	rs7307171	C	T	0.563	-0.0227189	0.00359853	2.70E-10	133471	0.0003	33.90
12	111323939	rs7311323	G	A	0.206	-0.134029	0.00442456	1.50E-201	133471	0.0059	789.55
12	113479646	rs4767056	C	T	0.460	0.0349588	0.0035743	1.40E-22	133471	0.0006	81.07
12	121420807	rs1183910	G	A	0.529	0.0925436	0.00344833	1.20E-158	133471	0.0043	572.10
12	132336077	rs117299843	C	T	0.697	-0.023462	0.00385038	1.10E-09	133471	0.0002	31.05
14	103571837	rs78702790	G	C	0.779	0.0840566	0.00419324	2.20E-89	133471	0.0024	325.77
15	39921132	rs1876852	G	A	0.633	-0.0205764	0.00360214	1.10E-08	133471	0.0002	26.26
15	60883281	rs339969	C	A	0.094	-0.0517593	0.00596091	3.90E-18	133471	0.0005	60.65
15	73956856	rs55868793	T	G	0.519	-0.0237526	0.00349363	1.10E-11	133471	0.0003	37.61
16	80497601	rs4581712	C	A	0.625	-0.0205104	0.00358419	1.00E-08	133471	0.0002	26.32
17	36077863	rs12951345	A	C	0.692	-0.0208361	0.00380349	4.30E-08	133471	0.0002	24.71
18	56096214	rs4245267	C	A	0.178	0.0573448	0.00454066	1.50E-36	133471	0.0010	128.65
20	39797465	rs753381	T	C	0.152	-0.0280412	0.00481736	5.90E-09	133471	0.0002	27.12
21	30513854	rs11330686	G	A	0.784	-0.0297709	0.00439438	1.20E-11	133471	0.0003	40.03
21	46271452	rs235314	C	T	0.594	0.0215401	0.0035482	1.30E-09	133471	0.0002	29.88

Abbreviations: CHR, chromosome; POS, position; SNP, single-nucleotide polymorphism; EA, effect allele; NEA, non-effect allele; EAF, effect allele frequency; N, the sample size of the genome-wide association study from which the genetic variants were selected.

Supplementary Table 9. Results where robust conclusions cannot be drawn of the second round two-sample Mendelian randomization analyses.

Exposure	Outcome	SNPs	IVW				MR-Egger						WM	
			β (95% CI)	*P* value	Cochran Q statistics (df)	*P* value	β (95% CI)	*P* value	Intercept (Se)	*P* value	Cochran Q statistics (df)	*P* value	β (95% CI)	*P* value
Ischemic stroke	SCLs	3	1.099 (-0.0004 2.199)	0.050	14.449 (2)	0.001	4.458 (2.221 6.695)	0.160	-0.285 (0.095)	0.204	1.431 (1)	0.232	0.728 (0.060 1.395)	0.033
Albumin (Alb)		20	0.197 (-0.261 0.656)	0.399	25.633 (19)	0.141	-1.014 (-1.895 -0.132)	0.037	0.054 (0.018)	0.007	16.564 (18)	0.553	-0.155 (-0.720 0.411)	0.592
Alanine aminotransferase (ALT)		19	0.619 (0.057 1.181)	0.031	21.612 (18)	0.250	-0.673 (-2.698 1.351)	0.523	0.039 (0.030)	0.211	19.657 (17)	0.292	0.624 (-0.151 1.400)	0.114
Body mass index (BMI)		57	0.363 (0.038 0.688)	0.029	49.009 (56)	0.735	0.612 (-0.362 1.587)	0.223	-0.007 (0.013)	0.597	48.726 (55)	0.712	0.320 (-0.193 0.832)	0.221
Mean corpuscular volume (MCV)		88	0.150 (-0.028 0.328)	0.098	105.417 (87)	0.087	0.375 (0.034 0.715)	0.034	-0.012 (0.008)	0.134	102.685 (86)	0.106	0.111 (-0.159 0.381)	0.421
Neutrophil count (Neutro)		25	-0.297 (-0.646 0.051)	0.095	35.940 (24)	0.056	-1.052 (-1.902 -0.202)	0.024	0.041 (0.022)	0.071	31.108 (23)	0.120	-0.003 (-0.429 0.424)	0.991
Red blood cell count (RBC)		63	-0.341 (-0.622 -0.060)	0.017	79.342 (62)	0.068	-0.611 (-1.180 -0.042)	0.039	0.011 (0.010)	0.289	77.884 (61)	0.071	-0.140 (-0.572 0.292)	0.524
White blood cell count (WBC)		53	-0.217 (-0.496 0.061)	0.127	49.045 (52)	0.591	-0.710 (-1.361 -0.059)	0.037	0.020 (0.012)	0.107	46.354 (51)	0.658	-0.231 (-0.683 0.221)	0.317

Abbreviations: SCLs, serum copper levels; MR, Mendelian randomization; SNPs, number of single-nucleotide polymorphism used as instrumental variables; IVW, inverse-variance weighted; WM, weighted median.

Supplementary Table 10. 16 exposure-outcome pairs that may have causal relationships from the third round two-sample Mendelian randomization analyses.

Exposure	Outcome	SNPs	IVW				MR-Egger						WM	
			OR (95% CI)	*P* value	Cochran Q statistics (df)	*P* value	OR (95% CI)	*P* value	Intercept (Se)	*P* value	Cochran Q statistics (df)	*P* value	OR (95% CI)	*P* value
ECLs	Adrenergics, inhalants	11	0.987 (0.954 1.021)	0.445	9.903 (10)	0.449	0.888 (0.808 0.975)	0.035	0.021 (0.009)	0.043	4.330 (9)	0.888	0.980 (0.934 1.028)	0.409
	Drugs affecting bone structure and mineralization	11	0.923 (0.869 0.981)	0.009	8.368 (10)	0.593	0.916 (0.775 1.084)	0.334	0.001 (0.016)	0.927	8.359 (9)	0.498	0.940 (0.866 1.021)	0.141
	Vasodilators used in cardiac diseases	11	0.967 (0.888 1.052)	0.431	13.772 (10)	0.184	0.781 (0.639 0.954)	0.038	0.042 (0.019)	0.052	8.748 (9)	0.461	0.950 (0.853 1.057)	0.343
	Acute hepatitis by Hepatitis A virus	12	2.191 (1.024 4.689)	0.043	17.994 (11)	0.082	1.422 (0.256 7.899)	0.696	0.097 (0.174)	0.590	17.454 (10)	0.065	4.317 (1.766 10.549)	0.001
	Carpal tunnel syndrome	12	1.041 (1.007 1.075)	0.018	6.792 (11)	0.816	1.024 (0.968 1.083)	0.426	0.005 (0.007)	0.507	6.318 (10)	0.788	1.033 (0.988 1.080)	0.153
	Compression fracture	12	0.841 (0.748 0.945)	0.004	5.706 (11)	0.892	0.862 (0.668 1.113)	0.283	-0.006 (0.026)	0.831	5.658 (10)	0.843	0.807 (0.693 0.941)	0.006
	Depression	12	1.052 (1.010 1.095)	0.015	15.383 (11)	0.166	1.064 (1.000 1.131)	0.078	-0.004 (0.009)	0.635	15.023 (10)	0.131	1.055 (1.001 1.112)	0.046
	Food allergy	12	1.766 (1.111 2.809)	0.016	16.954 (11)	0.109	2.366 (0.797 7.028)	0.152	-0.064 (0.109)	0.571	16.391 (10)	0.089	1.468 (0.820 2.630)	0.197
	Hearing loss, difficulty in hearing	12	1.007 (0.963 1.053)	0.767	19.825 (11)	0.048	0.941 (0.893 0.991)	0.044	0.022 (0.007)	0.008	8.783 (10)	0.553	0.973 (0.927 1.021)	0.265
	Neuropathic bladder	12	1.093 (0.996 1.200)	0.062	11.499 (11)	0.402	1.223 (1.046 1.431)	0.030	-0.032 (0.018)	0.114	8.499 (10)	0.580	1.146 (1.005 1.307)	0.041
	Ovarian cyst	12	1.047 (1.005 1.090)	0.027	9.243 (11)	0.600	1.062 (0.996 1.133)	0.097	-0.005 (0.008)	0.584	8.923 (10)	0.539	1.056 (0.998 1.117)	0.058
	Spinal canal stenosis	12	1.063 (1.018 1.110)	0.005	8.683 (11)	0.651	1.104 (1.030 1.183)	0.018	-0.012 (0.009)	0.199	6.795 (10)	0.745	1.079 (1.011 1.152)	0.022
	Stable angina pectoris	12	0.971 (0.926 1.018)	0.217	15.068 (11)	0.179	0.918 (0.830 1.016)	0.130	0.012 (0.010)	0.256	13.157 (10)	0.215	0.943 (0.895 0.993)	0.027
	Systemic lupus erythematosus	12	0.849 (0.721 0.998)	0.048	8.702 (11)	0.649	0.820 (0.627 1.071)	0.176	0.010 (0.032)	0.755	8.599 (10)	0.571	0.818 (0.653 1.024)	0.079
	Ulcerative colitis	12	1.062 (1.002 1.125)	0.042	8.113 (11)	0.703	1.037 (0.943 1.141)	0.471	0.007 (0.012)	0.559	7.747 (10)	0.654	1.043 (0.966 1.127)	0.280
	Urticaria	12	0.914 (0.803 1.042)	0.179	13.329 (11)	0.272	0.766 (0.637 0.921)	0.018	0.059 (0.024)	0.034	7.293 (10)	0.698	0.875 (0.732 1.046)	0.143

Abbreviations: ECLs, erythrocytes copper levels; MR, Mendelian randomization; SNPs, number of single-nucleotide polymorphism used as instrumental variables; IVW, inverse-variance weighted; WM, weighted median.
Note: * OR and 95% confidence interval are replaced by β and 95% confidence interval.

Supplementary Table 11. Results where robust conclusions cannot be drawn of the third round two-sample Mendelian randomization analyses.

Exposure	Outcome	SNPs	IVW				MR-Egger						WM	
			OR (95% CI)	*P* value	Cochran Q statistics (df)	*P* value	OR (95% CI)	*P* value	Intercept (Se)	*P* value	Cochran Q statistics (df)	*P* value	OR (95% CI)	*P* value
ECLs	Adrenergics, inhalants	11	0.987 (0.954 1.021)	0.445	9.903 (10)	0.449	0.888 (0.808 0.975)	0.035	0.021 (0.009)	0.043	4.330 (9)	0.888	0.980 (0.934 1.028)	0.409
	Drugs affecting bone structure and mineralization	11	0.923 (0.869 0.981)	0.009	8.368 (10)	0.593	0.916 (0.775 1.084)	0.334	0.001 (0.016)	0.927	8.359 (9)	0.498	0.940 (0.866 1.021)	0.141
	Vasodilators used in cardiac diseases	11	0.967 (0.888 1.052)	0.431	13.772 (10)	0.184	0.781 (0.639 0.954)	0.038	0.042 (0.019)	0.052	8.748 (9)	0.461	0.950 (0.853 1.057)	0.343
	Acute hepatitis by Hepatitis A virus	12	2.191 (1.024 4.689)	0.043	17.994 (11)	0.082	1.422 (0.256 7.899)	0.696	0.097 (0.174)	0.590	17.454 (10)	0.065	4.317 (1.766 10.549)	0.001
	Depression	12	1.052 (1.010 1.095)	0.015	15.383 (11)	0.166	1.064 (1.000 1.131)	0.078	-0.004 (0.009)	0.635	15.023 (10)	0.131	1.055 (1.001 1.112)	0.046
	Food allergy	12	1.766 (1.111 2.809)	0.016	16.954 (11)	0.109	2.366 (0.797 7.028)	0.152	-0.064 (0.109)	0.571	16.391 (10)	0.089	1.468 (0.820 2.630)	0.197
	Hearing loss, difficulty in hearing	12	1.007 (0.963 1.053)	0.767	19.825 (11)	0.048	0.941 (0.893 0.991)	0.044	0.022 (0.007)	0.008	8.783 (10)	0.553	0.973 (0.927 1.021)	0.265
	Neuropathic bladder	12	1.093 (0.996 1.200)	0.062	11.499 (11)	0.402	1.223 (1.046 1.431)	0.030	-0.032 (0.018)	0.114	8.499 (10)	0.580	1.146 (1.005 1.307)	0.041
	Ovarian cyst	12	1.047 (1.005 1.090)	0.027	9.243 (11)	0.600	1.062 (0.996 1.133)	0.097	-0.005 (0.008)	0.584	8.923 (10)	0.539	1.056 (0.998 1.117)	0.058
	Spinal canal stenosis	12	1.063 (1.018 1.110)	0.005	8.683 (11)	0.651	1.104 (1.030 1.183)	0.018	-0.012 (0.009)	0.199	6.795 (10)	0.745	1.079 (1.011 1.152)	0.022
	Stable angina pectoris	12	0.971 (0.926 1.018)	0.217	15.068 (11)	0.179	0.918 (0.830 1.016)	0.130	0.012 (0.010)	0.256	13.157 (10)	0.215	0.943 (0.895 0.993)	0.027
	Systemic lupus erythematosus	12	0.849 (0.721 0.998)	0.048	8.702 (11)	0.649	0.820 (0.627 1.071)	0.176	0.010 (0.032)	0.755	8.599 (10)	0.571	0.818 (0.653 1.024)	0.079
	Ulcerative colitis	12	1.062 (1.002 1.125)	0.042	8.113 (11)	0.703	1.037 (0.943 1.141)	0.471	0.007 (0.012)	0.559	7.747 (10)	0.654	1.043 (0.966 1.127)	0.280
	Urticaria	12	0.914 (0.803 1.042)	0.179	13.329 (11)	0.272	0.766 (0.637 0.921)	0.018	0.059 (0.024)	0.034	7.293 (10)	0.698	0.875 (0.732 1.046)	0.143

Abbreviations: ECLs, erythrocytes copper levels; MR, Mendelian randomization; SNPs, number of single-nucleotide polymorphism used as instrumental variables; IVW, inverse-variance weighted; WM, weighted median.
Note: * OR and 95% confidence interval are replaced by β and 95% confidence interval.

Supplementary Table 12. Summary of the genetic instrumental variables of serum copper levels.

Metals	CHR	POS (hg19)	SNP	EA	NEA	EAF	BETA	SE	*P*	N	R^2	F statistic	Q	I^2
	3	125651240	rs114692803	C	A	0.113	0.208	0.047	9.91E-06	2483	0.0087	21.74	0.7813	0
	3	148940672	rs35691438	C	T	0.325	0.187	0.031	1.63E-09	2483	0.0153	38.55	0.4967	0
	6	31248626	rs369578889	C	T	0.017	0.488	0.107	5.55E-06	2483	0.0082	20.40	0.8261	0
	10	5724382	rs10796180	A	G	0.249	0.158	0.034	2.55E-06	2483	0.0094	23.47	0.9332	0
	12	70996021	rs11178321	G	T	0.239	-0.152	0.033	4.39E-06	2483	0.0084	20.90	0.385	0
SCLs	12	112241766	rs671	A	G	0.252	-0.207	0.033	2.21E-10	2483	0.0161	40.65	0.3499	0
	14	25709178	rs66541674	A	G	0.174	-0.188	0.039	1.78E-06	2483	0.0101	25.43	0.4815	0
	14	95763173	rs34703907	G	A	0.269	0.141	0.032	7.71E-06	2483	0.0079	19.65	0.5037	0
	15	86923959	rs16976566	T	C	0.040	-0.373	0.079	2.32E-06	2483	0.0108	27.01	0.6005	0
	17	31793131	rs7209964	A	C	0.234	0.148	0.033	8.75E-06	2483	0.0078	19.54	0.8356	0
	19	14951605	rs3752192	G	A	0.111	0.205	0.045	4.11E-06	2483	0.0083	20.83	0.1877	42.39

Abbreviations: SCLs, serum copper levels; CHR, chromosome; POS, position; SNP, single-nucleotide polymorphism; EA, effect allele; NEA, non-effect allele; EAF, effect allele frequency; N, the sample size of the genome-wide association studies or meta-analysis from which the genetic variants were selected; Q, p-value for Cochran's Q statistic; I^2, heterogeneity index (0-100 scale); R^2, proportion of the transformed serum copper variance explained by the selected genetic variants.

Supplementary Table 13. Summary of the genetic instrumental variables of erythrocytes copper levels.

Metals	CHR	POS (hg19)	SNP	EA	NEA	EAF	BETA	SE	*P*	N	R^2	F statistic
ECLs	1	151344741	rs2769264	G	T	0.180	0.313	0.034	2.63E-20	2603	0.0289	77.46
	1	151355858	rs2769270	A	G	0.143	-0.349	0.046	4.36E-14	2603	0.0299	80.04
	1	3691528	rs1175550	A	G	0.770	-0.198	0.032	5.03E-10	2603	0.0139	36.63
	2	119354478	rs572585	C	T	0.748	-0.137	0.031	9.13E-06	2603	0.0071	18.54
	3	142352978	rs13074172	A	G	0.562	-0.121	0.027	7.16E-06	2603	0.0072	18.88
	4	113948790	rs10014072	A	G	0.279	0.164	0.034	1.13E-06	2603	0.0108	28.45
	5	84583769	rs12153606	G	T	0.810	0.159	0.034	2.50E-06	2603	0.0078	20.40
	6	66929048	rs3857536	C	T	0.438	0.129	0.028	4.08E-06	2603	0.0082	21.48
	7	27359068	rs764560	C	T	0.654	0.128	0.028	6.42E-06	2603	0.0074	19.43
	8	139727981	rs9324493	A	G	0.866	0.177	0.039	5.94E-06	2603	0.0073	19.05
	12	76064528	rs12582659	C	T	0.011	1.262	0.27	2.86E-06	2603	0.0347	93.37
	16	56381277	rs7206796	C	T	0.794	0.152	0.034	9.26E-06	2603	0.0076	19.81

Abbreviations: ECLs, erythrocytes copper levels; CHR, chromosome; POS, position; SNP, single-nucleotide polymorphism; EA, effect allele; NEA, non-effect allele; EAF, effect allele frequency; N, the sample size of the genome-wide association studies or meta-analysis from which the genetic variants were selected.

Supplementary Figure 1. The Manhattan, Quantile-Quantile and LocusZoom plots of GWAS on serum copper levels in FAMHES.

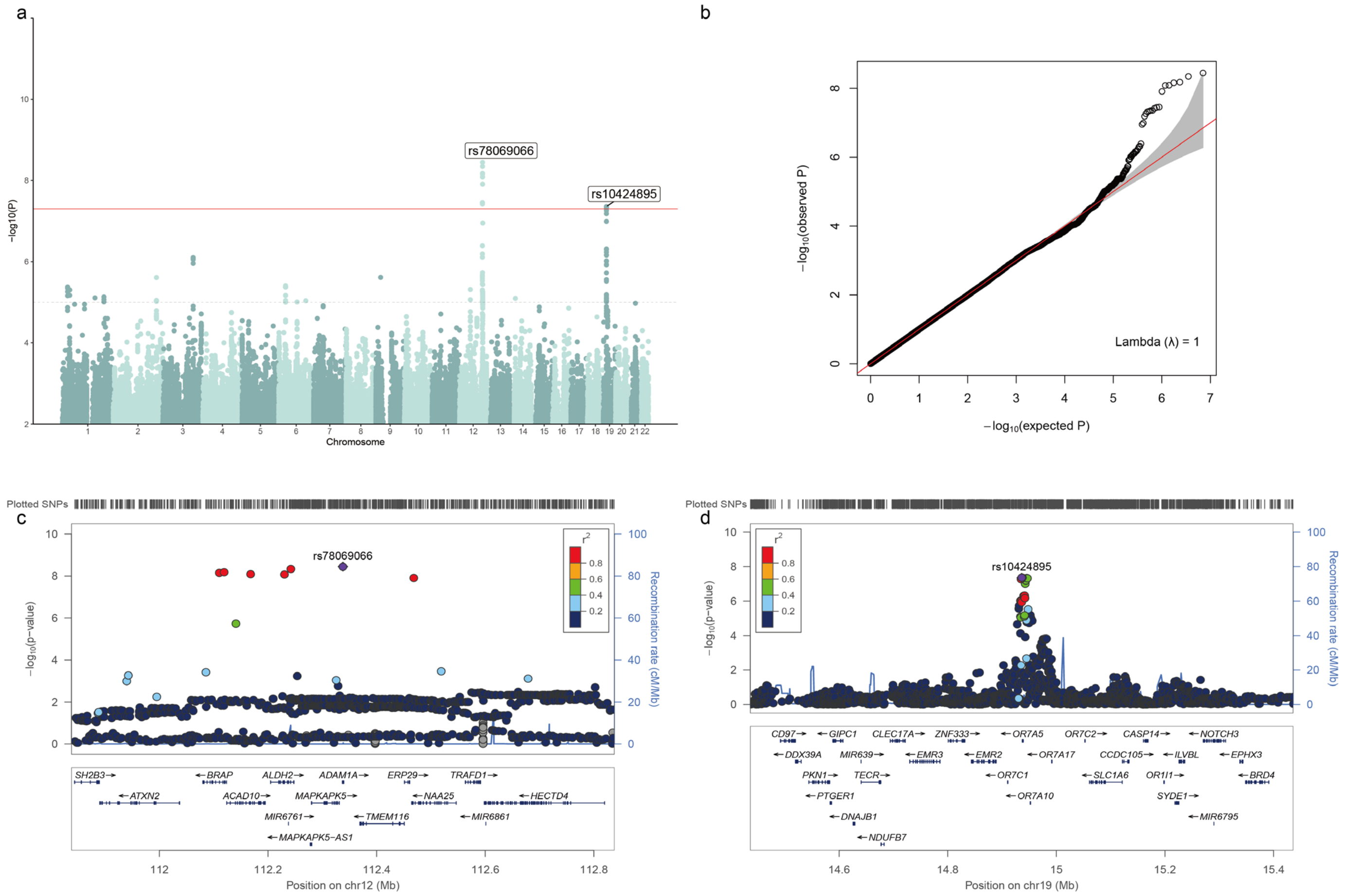

a: The X-axis presents the genomic position, while the Y-axis presents the -log$_{10}$(P) of SNPs. The red solid line is the genome-wide significance threshold (P = 5.0 × 10^{-8}), and the black dashed line is the genome-wide suggestive threshold (P = 1.0 × 10^{-5}). **b:** The QQ plot shows the deviation of the observed from the expected P-values under the null hypothesis of no association. **c:** LocusZoom plot for gene *ADAM1A*. Purple diamond indicates SNP at the locus with the strongest association evidence (rs78069066) and the r^2 (degree of linkage disequilibrium) is 1. **d:** LocusZoom plot for gene *OR7A5*. Purple diamond indicates SNP at the locus with the strongest association evidence (rs10424895) and the r^2 (degree of linkage disequilibrium) is 1.

Supplementary Figure 2. The Manhattan, Quantile-Quantile and LocusZoom plot of GWAS on plasma iron levels in MEWHC.

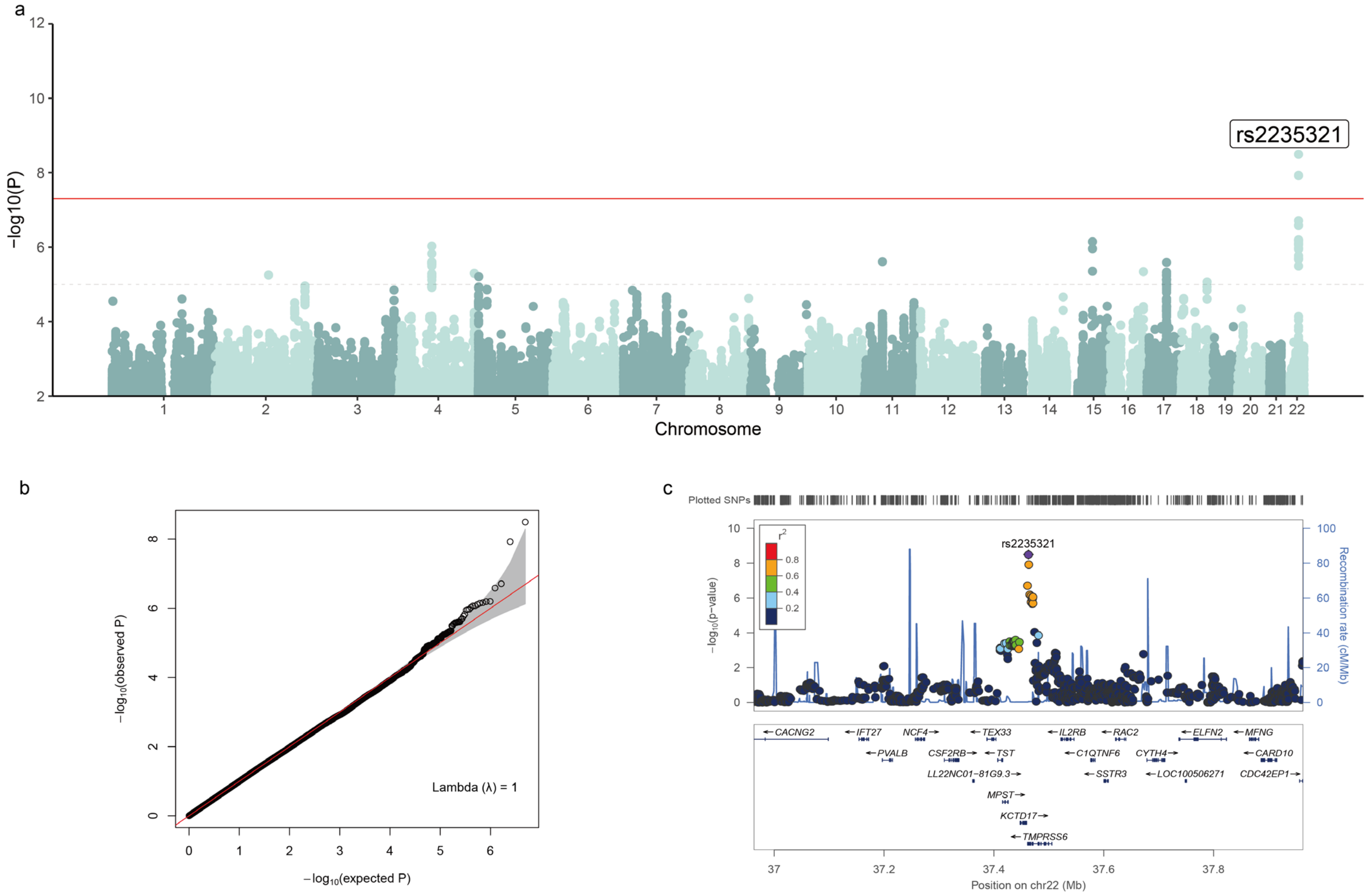

a: The X-axis presents the genomic position, while the Y-axis presents the -$\log_{10}(P)$ of SNPs. The red solid line is the genome-wide significance threshold ($P = 5.0 \times 10^{-8}$), and the black dashed line is the genome-wide suggestive threshold ($P = 1.0 \times 10^{-5}$). **b:** The QQ plot shows the deviation of the observed from the expected *P*-values under the null hypothesis of no association. **c:** LocusZoom plot for gene *TMPRSS6*. Purple diamond indicates SNP at the locus with the strongest association evidence (rs2235321) and the r^2 (degree of linkage disequilibrium) is 1.

Supplementary Figure 3. The gene expression heatmap of 14 potential causal genes.

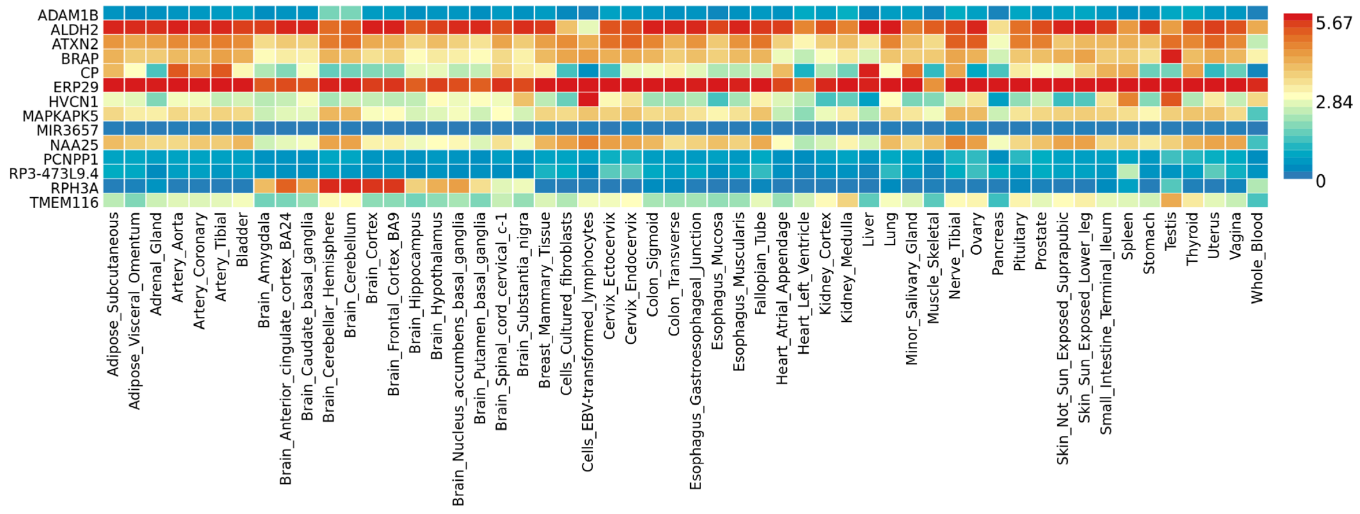

Supplementary Figure 4. Plots of leave-one-out analyses for four results where robust conclusions cannot be drawn in the first round two-sample Mendelian randomization analyses.

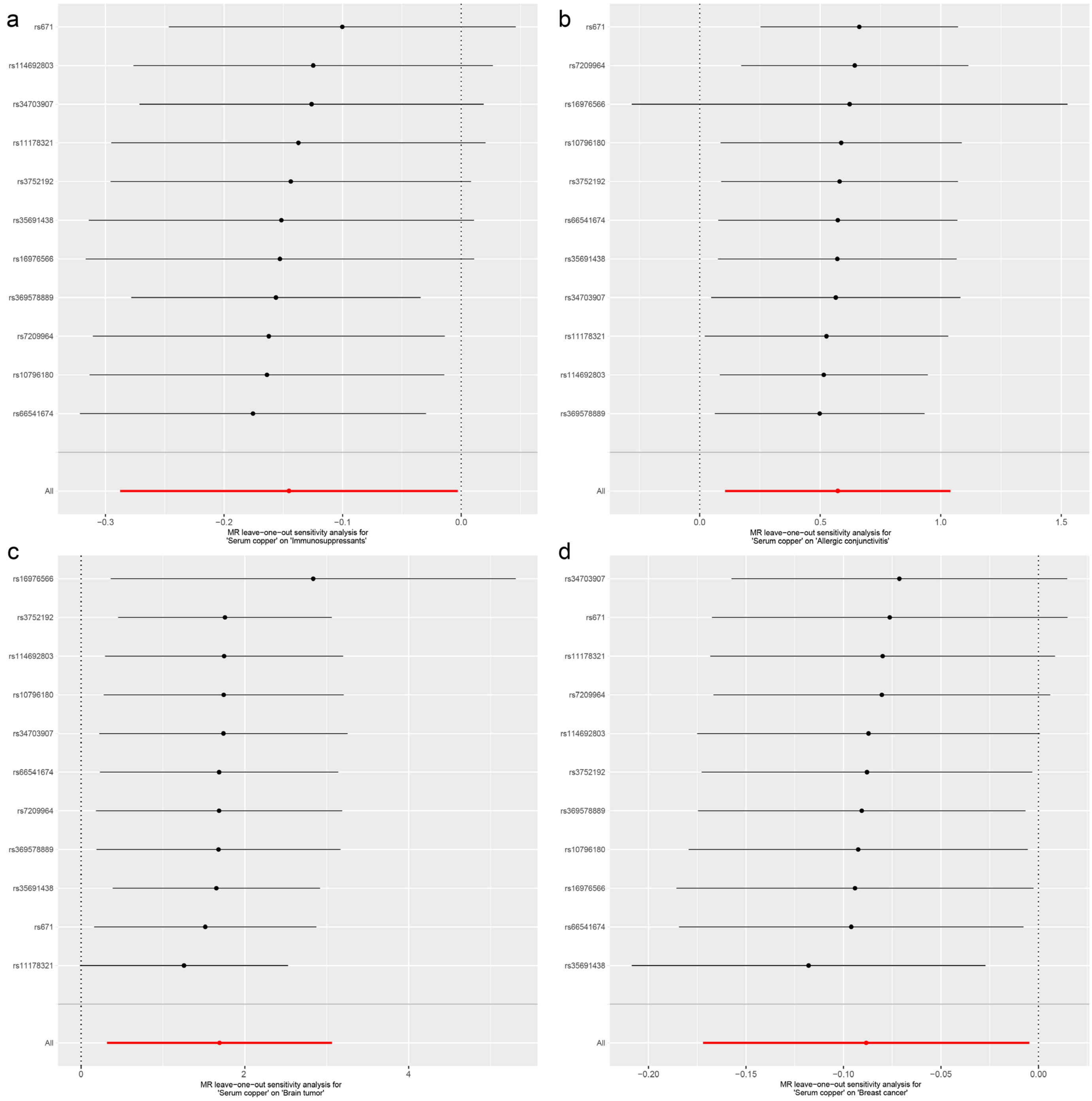

The error bars indicate the 95% confidence interval. **a:** usage of immunosuppressants. **b:** allergic conjunctivitis. **c:** brain tumor. **d:** breast cancer. MR Mendelian randomization.

Supplementary Figure 5. Plots of leave-one-out analyses for four results where robust conclusions cannot be drawn in the first round two-sample Mendelian randomization analyses.

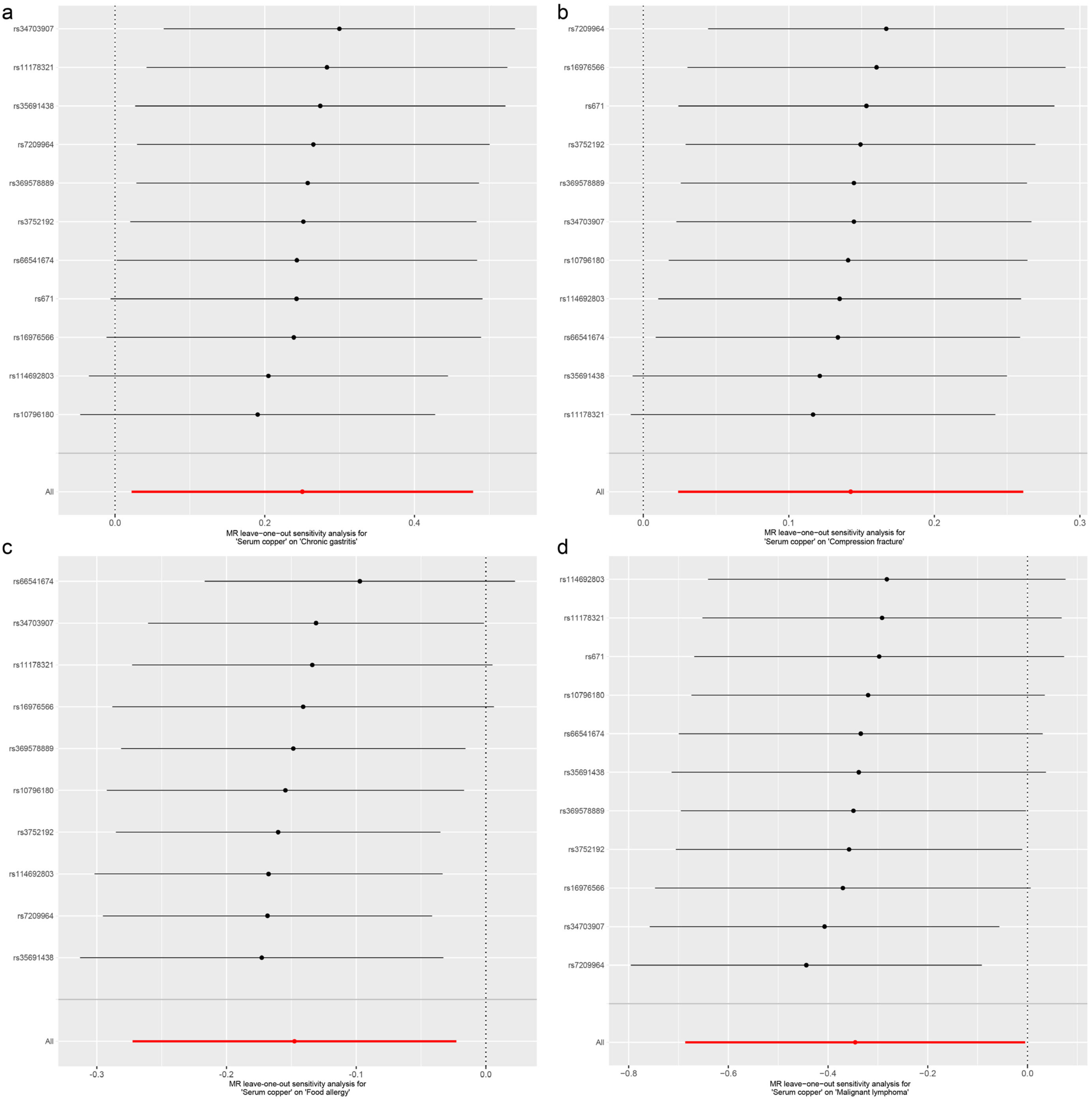

The error bars indicate the 95% confidence interval. **a:** chronic gastritis. **b:** compression fracture. **c:** food allergy. **d:** malignant lymphoma. MR Mendelian randomization.

Supplementary Figure 6. Plots of leave-one-out analyses for three results where robust conclusions cannot be drawn in the first round two-sample Mendelian randomization analyses.

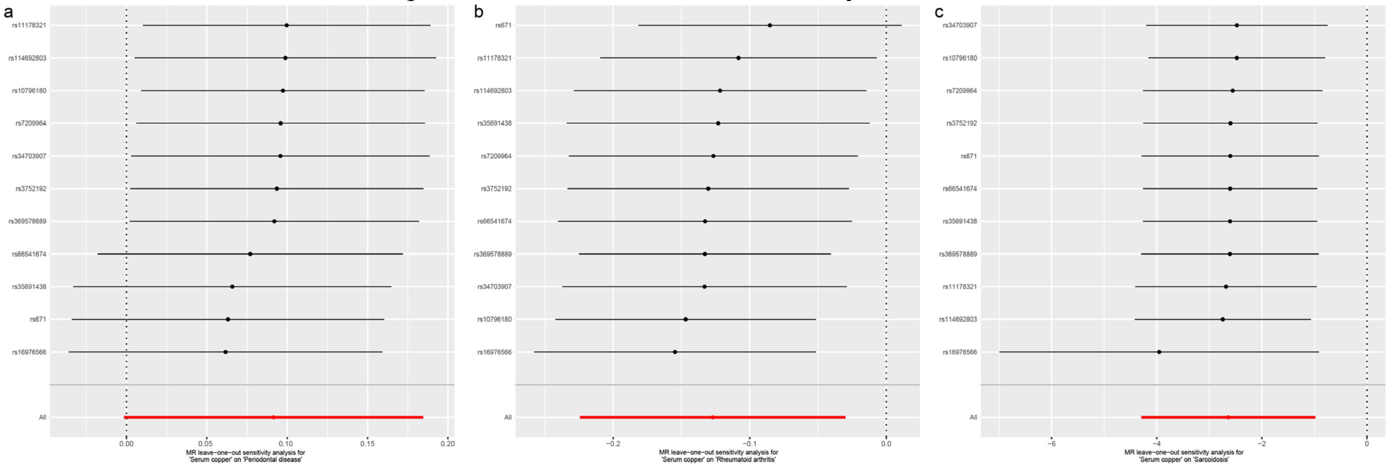

The error bars indicate the 95% confidence interval. **a:** periodontal disease**. b:** rheumatoid arthritis**. c:** sarcoidosis. MR Mendelian randomization.

Supplementary Figure 7. Plots of leave-one-out analyses for two results where robust conclusions cannot be drawn in the first round two-sample Mendelian randomization analyses.

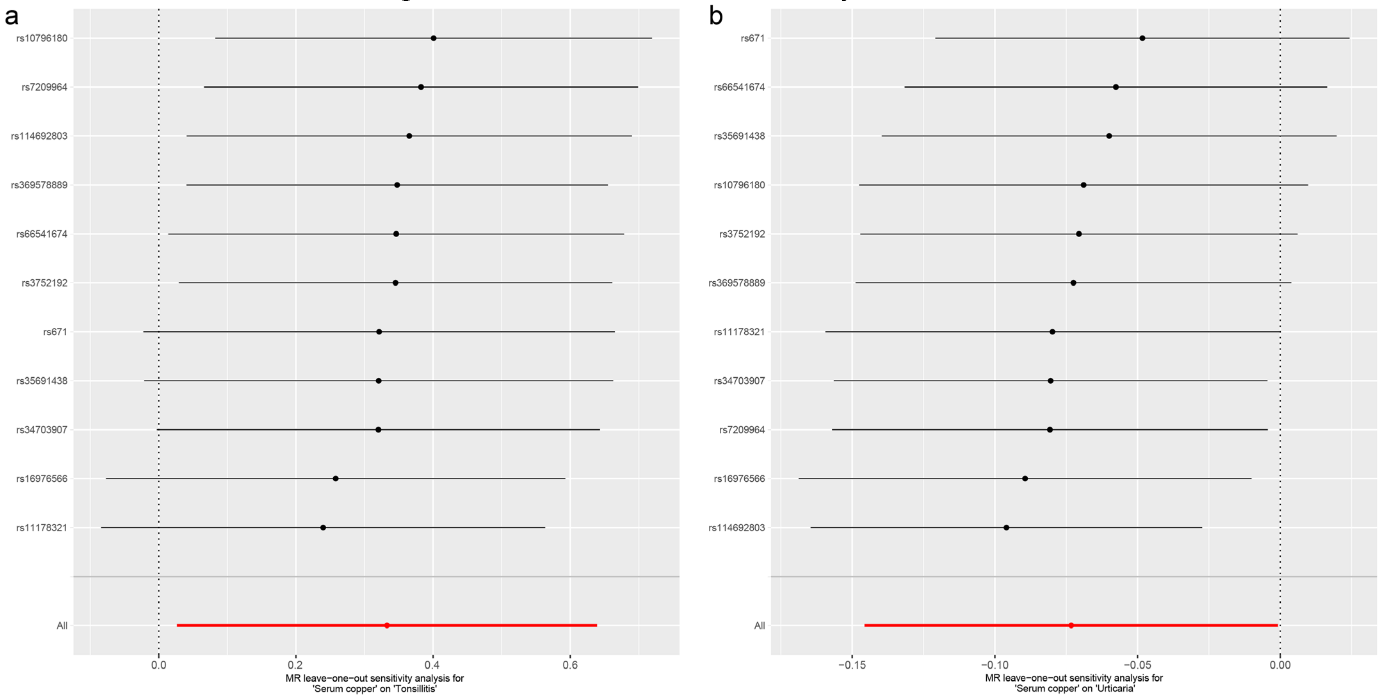

The error bars indicate the 95% confidence interval. **a:** tonsillitis. **b:** urticaria. MR Mendelian randomization.

Supplementary Figure 8. Plots of leave-one-out analyses for four results where robust conclusions cannot be drawn in the second round two-sample Mendelian randomization analyses.

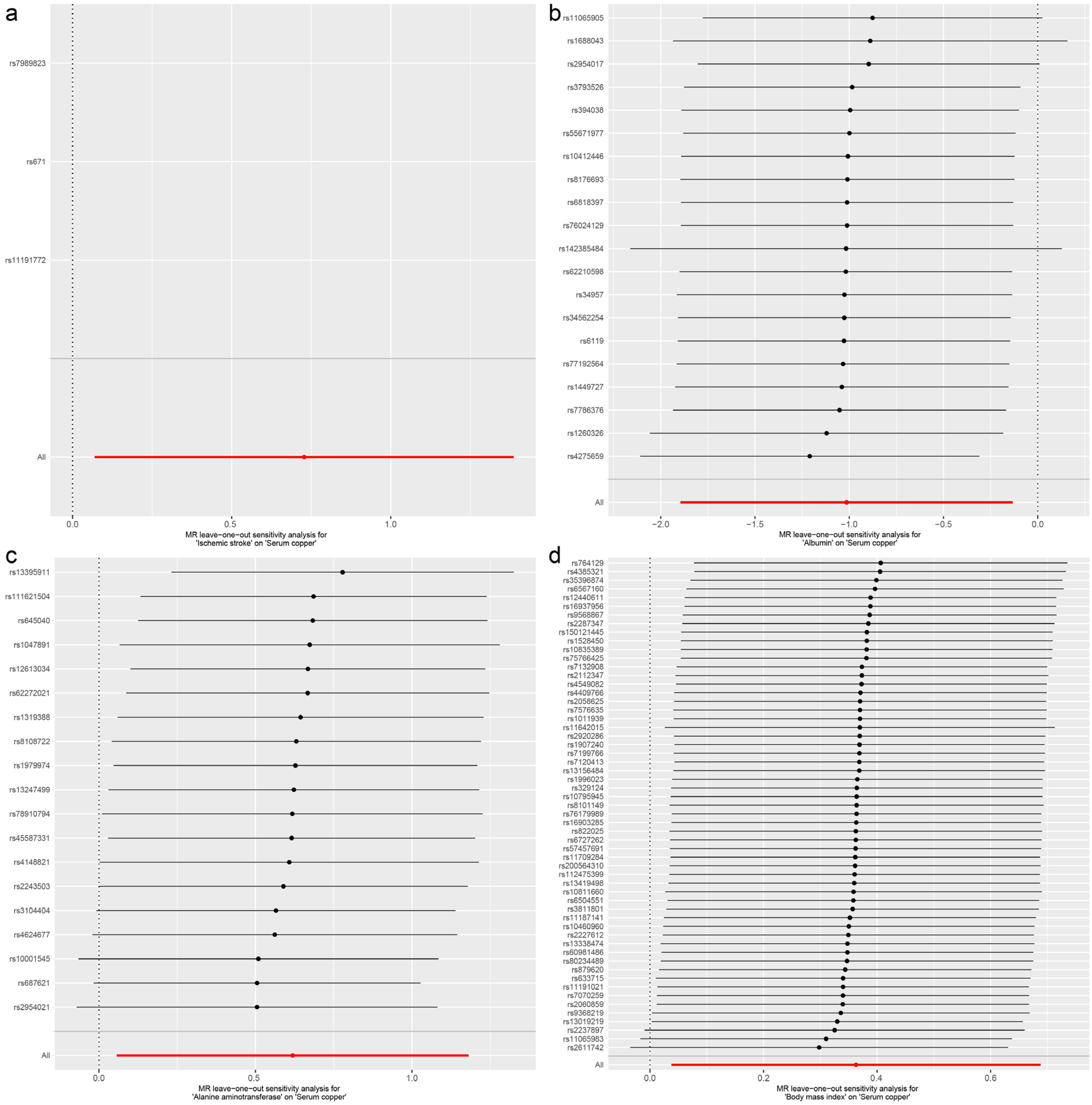

The error bars indicate the 95% confidence interval. **a:** ischemic stroke. **b:** albumin. **c:** alanine aminotransferase. **d:** body mass index. MR Mendelian randomization.

Supplementary Figure 9. Plots of leave-one-out analyses for four results where robust conclusions cannot be drawn in the second round two-sample Mendelian randomization analyses.

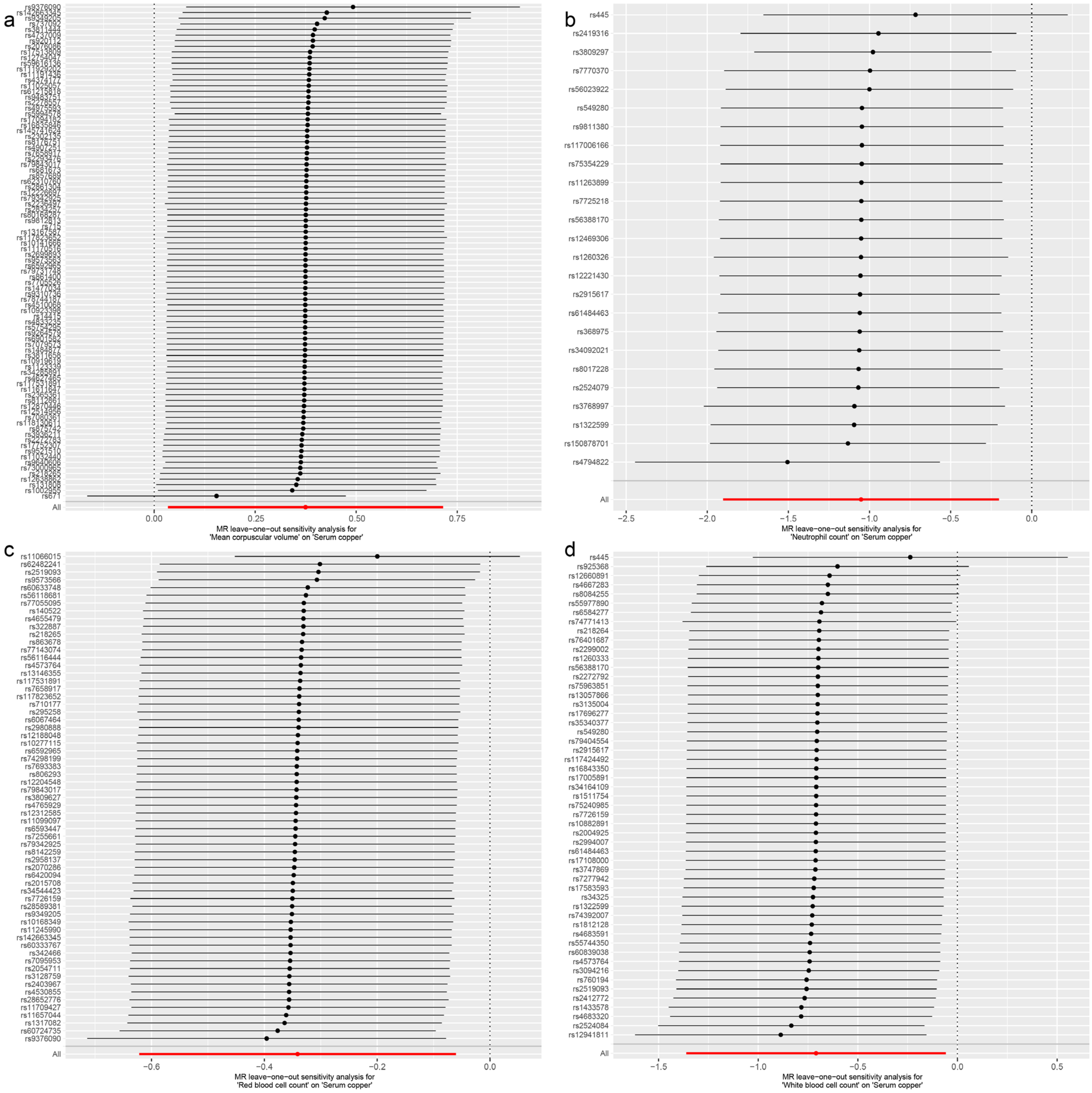

The error bars indicate the 95% confidence interval. **a:** mean corpuscular volume. **b:** neutrophil count. **c:** red blood cell count. **d:** white blood cell count. MR Mendelian randomization.

Supplementary Figure 10. Plots of leave-one-out analyses for four results where robust conclusions cannot be drawn in the third round two-sample Mendelian randomization analyses.

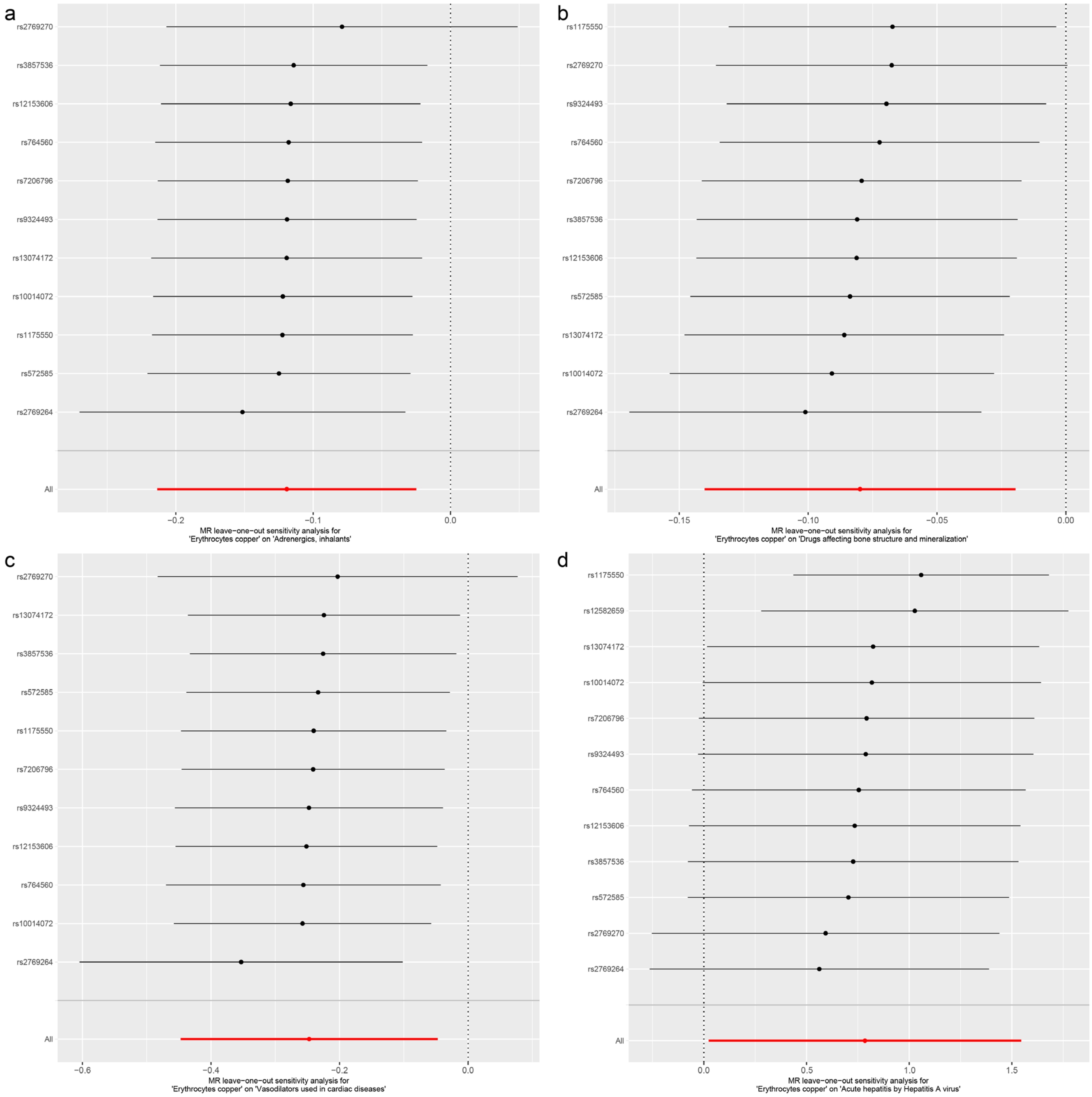

The error bars indicate the 95% confidence interval. **a:** usage of adrenergics, inhalants. **b:** usage of drugs affecting bone structure and mineralization. **c:** usage of vasodilators used in cardiac diseases. **d:** acute hepatitis by hepatitis A virus. MR Mendelian randomization.

Supplementary Figure 11. Plots of leave-one-out analyses for four results where robust conclusions cannot be drawn in the third round two-sample Mendelian randomization analyses.

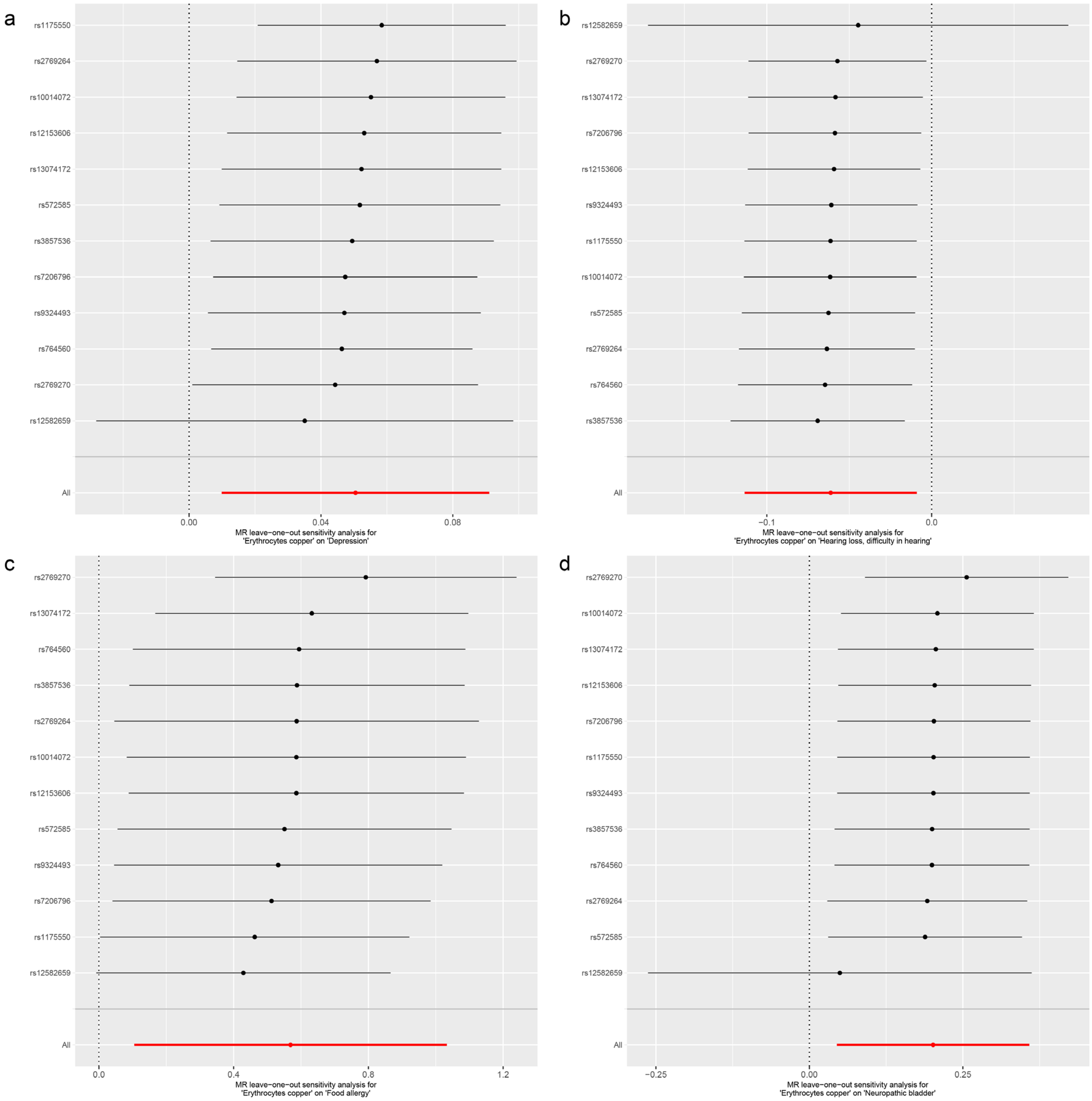

The error bars indicate the 95% confidence interval. **a:** depression. **b:** hearing loss, difficulty in hearing. **c:** food allergy. **d:** neuropathic bladder. MR Mendelian randomization.

Supplementary Figure 12. Plots of leave-one-out analyses for four results where robust conclusions cannot be drawn in the third round two-sample Mendelian randomization analyses.

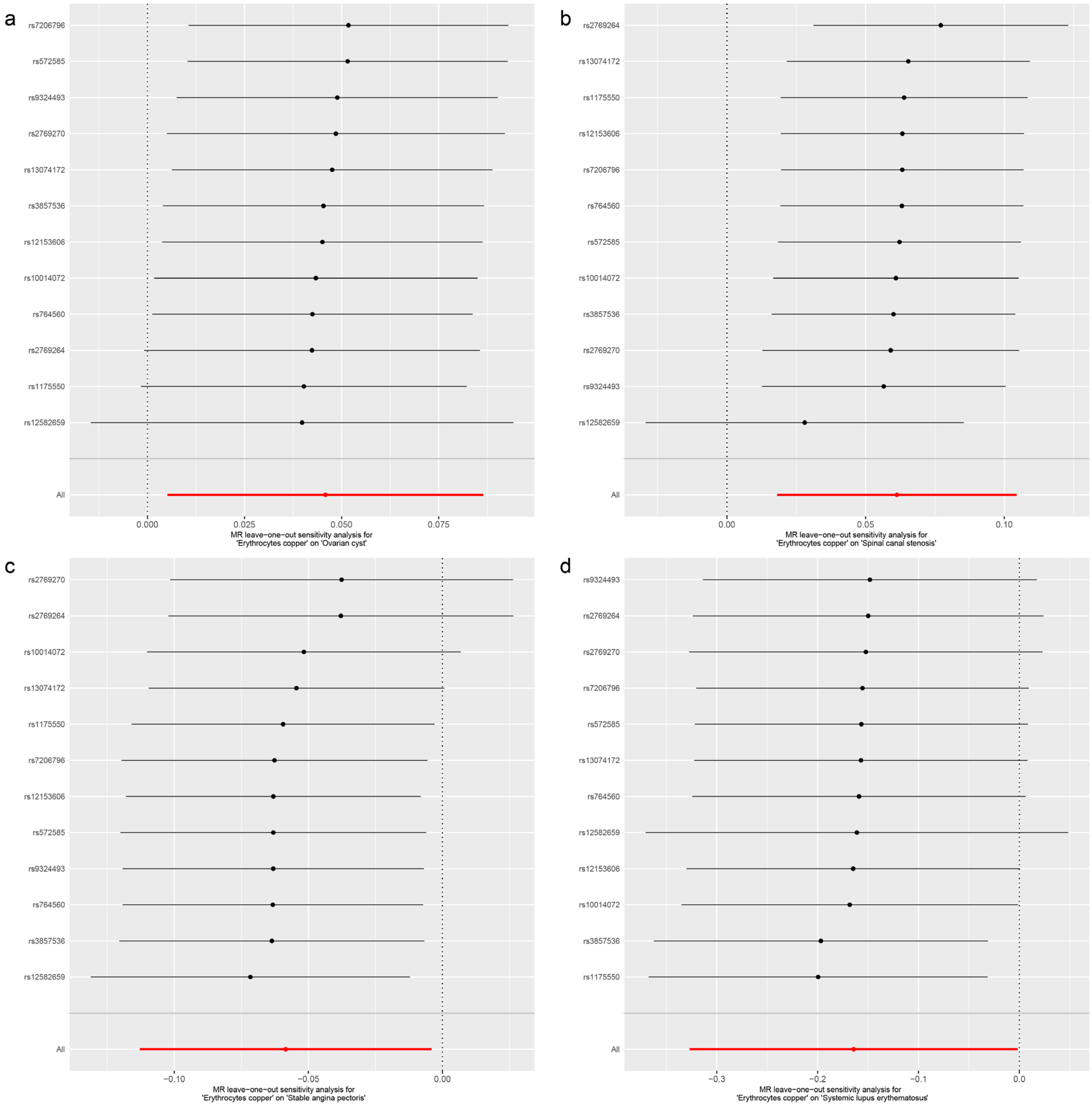

The error bars indicate the 95% confidence interval. **a:** ovarian cyst. **b:** spinal canal stenosis. **c:** stable angina pectoris. **d:** systemic lupus erythematosus. MR Mendelian randomization.

Supplementary Figure 13. Plots of leave-one-out analyses for two results where robust conclusions cannot be drawn in the third round two-sample Mendelian randomization analyses.

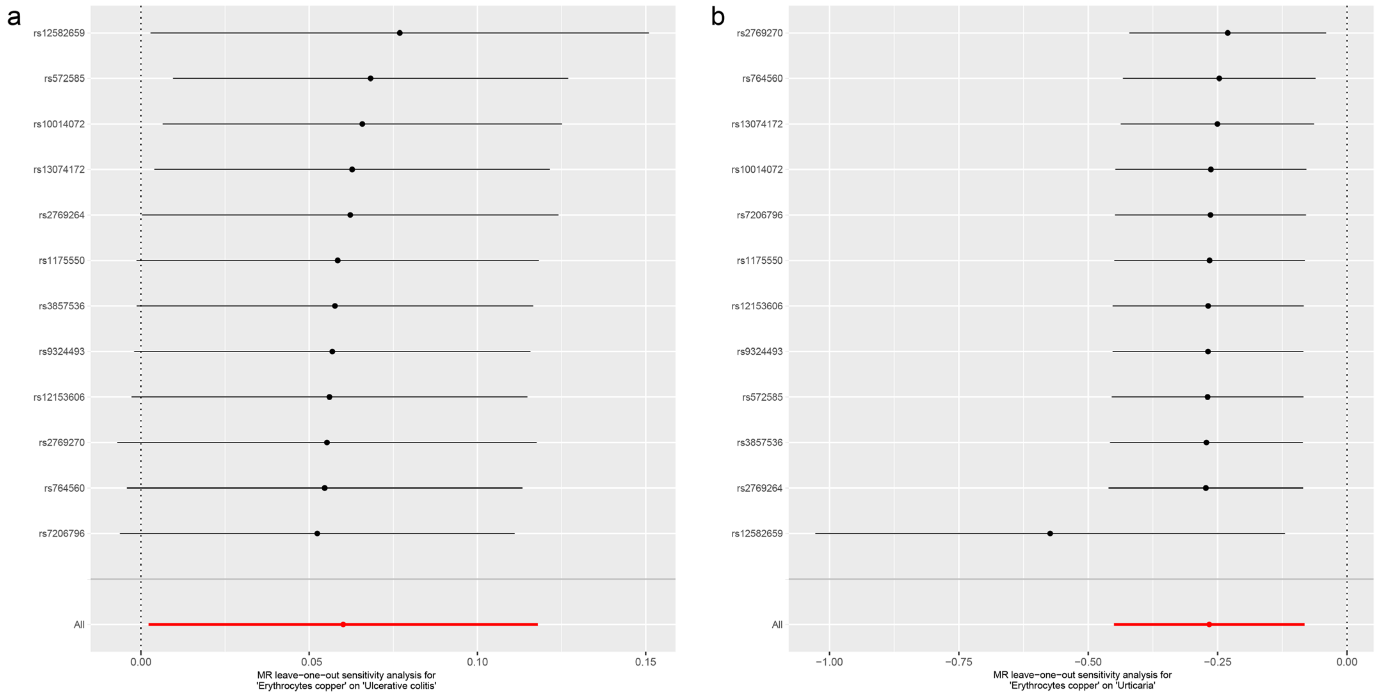

The error bars indicate the 95% confidence interval. **a:** ulcerative colitis. **b:** urticaria. MR Mendelian randomization.
