# Supplementary material for: Genome-wide association and Mendelian randomization study of blood copper levels and 213 deep phenotypes in humans
Source: Commun Biol. 2022 May 2;5:405. doi: 10.1038/s42003-022-03351-7 (PMC9061855; doi:10.1038/s42003-022-03351-7)
Supplement: Supplementary file 2 — Description of Additional Supplementary Files [file 42003_2022_3351_MOESM2_ESM.pdf]

# Description of Additional Supplementary Files

**File name:** Supplementary Data 1
**Description:** Suggestive SNPs (P < 1E-5) from GWASs on 21 serum/plasma metal levels in FAMHES and MEWHC.

**File name:** Supplementary Data 2
**Description:** Prioritized genes by functional mapping based on GWAS meta-analysis of serum copper levels.

**File name:** Supplementary Data 3
**Description:** Information of 213 deep phenotypes used in our two-sample Mendelian randomization analyses of the East Asian population.

**File name:** Supplementary Data 4
**Description:** Information of 174 deep phenotypes used in our two-sample Mendelian randomization analyses of the European population.
