# Supplementary material for: Genome-wide association and Mendelian randomization study of blood copper levels and 213 deep phenotypes in humans
Source: Commun Biol. 2022 May 2;5:405. doi: 10.1038/s42003-022-03351-7 (PMC9061855; doi:10.1038/s42003-022-03351-7)
Supplement: Supplementary file 7 — Reporting Summary [file 42003_2022_3351_MOESM7_ESM.pdf]

nature portfolio

Corresponding author(s): Xiaobo Yang

Last updated by author(s): Mar 29, 2022

Reporting Summary

Nature Portfolio wishes to improve the reproducibility of the work that we publish. This form provides structure for consistency and transparency in reporting. For further information on Nature Portfolio policies, see our Editorial Policies and the Editorial Policy Checklist.

Statistics

For all statistical analyses, confirm that the following items are present in the figure legend, table legend, main text, or Methods section.

n/a	Confirmed	
☐	☒	The exact sample size (*n*) for each experimental group/condition, given as a discrete number and unit of measurement
☐	☒	A statement on whether measurements were taken from distinct samples or whether the same sample was measured repeatedly
☐	☒	The statistical test(s) used AND whether they are one- or two-sided<br>*Only common tests should be described solely by name; describe more complex techniques in the Methods section.*
☐	☒	A description of all covariates tested
☐	☒	A description of any assumptions or corrections, such as tests of normality and adjustment for multiple comparisons
☐	☒	A full description of the statistical parameters including central tendency (e.g. means) or other basic estimates (e.g. regression coefficient) AND variation (e.g. standard deviation) or associated estimates of uncertainty (e.g. confidence intervals)
☐	☒	For null hypothesis testing, the test statistic (e.g. *F*, *t*, *r*) with confidence intervals, effect sizes, degrees of freedom and *P* value noted<br>*Give P values as exact values whenever suitable.*
☒	☐	For Bayesian analysis, information on the choice of priors and Markov chain Monte Carlo settings
☒	☐	For hierarchical and complex designs, identification of the appropriate level for tests and full reporting of outcomes
☐	☒	Estimates of effect sizes (e.g. Cohen's *d*, Pearson's *r*), indicating how they were calculated

Our web collection on statistics for biologists contains articles on many of the points above.

Software and code

Policy information about availability of computer code

Data collection	We used an inductively coupled plasma mass spectrometry (ICP-MS, Perkin Elmer NexION 350xX, USA) to measure the serum/plasma metal concentrations
Data analysis	Plink2, R 4.0.5, liftOverPlink, Snpflip, IBM SPSS Statistics 25

For manuscripts utilizing custom algorithms or software that are central to the research but not yet described in published literature, software must be made available to editors and reviewers. We strongly encourage code deposition in a community repository (e.g. GitHub). See the Nature Portfolio guidelines for submitting code & software for further information.

Data

Policy information about availability of data

All manuscripts must include a data availability statement. This statement should provide the following information, where applicable:

- Accession codes, unique identifiers, or web links for publicly available datasets
- A description of any restrictions on data availability
- For clinical datasets or third party data, please ensure that the statement adheres to our policy

The GWAS summary statistics of 213 deep phenotypes can be downloaded from the website (https://pheweb.jp/downloads). The GWAS summary statistics of serum/plasma metal levels have been provided to the NHGRI-EBI GWAS Catalog and the study accession numbers are GCST90100517, GCST90100518, GCST90100519, GCST90100520, GCST90100521, GCST90100522, GCST90100523, GCST90100524, GCST90100525, GCST90100526, GCST90100527, GCST90100528, GCST90100529, GCST90100530, GCST90100531, GCST90100532, GCST90100533, GCST90100534, GCST90100535, GCST90100536, GCST90100537, GCST90100538, GCST90100539, GCST90100540, GCST90100541, GCST90100542, GCST90100543, GCST90100544, GCST90100545, GCST90100546, GCST90100547, GCST90100548, GCST90100549, GCST90100550, GCST90100551, GCST90100552, GCST90100553, GCST90100554,

GCST90100555, GCST90100556, GCST90100557, GCST90100558. The source data for graphs and charts can be downloaded from Figshare (https://figshare.com) through the DOI 10.6084/m9.figshare.17696942. The datasets generated and analyzed during the current study are available in the Genome variation Map (GVM) of National Genomics Data Center (NGDC) (Accession Number: GVM000052). Please contact the corresponding author for more information if necessary.

Field-specific reporting

Please select the one below that is the best fit for your research. If you are not sure, read the appropriate sections before making your selection.

☒ Life sciences ☐ Behavioural & social sciences ☐ Ecological, evolutionary & environmental sciences

For a reference copy of the document with all sections, see nature.com/documents/nr-reporting-summary-flat.pdf

Life sciences study design

All studies must disclose on these points even when the disclosure is negative.

Sample size	After quality control, we totally included 2488 subjects in our genome-wide association studies.
Data exclusions	In the genotyping stage, we excluded individuals with (1) individuals call rate < 0.95, (2) genotypic and phenotypic sex mismatch, (3) heterozygosity rate deviating more than three standard deviations from the mean, (4) kinship coefficient > 0.0884, (5) missingness of phenotypes or covariates. We also excluded SNPs with (1) call rate < 0.95, (2) minor allele frequency (MAF) < 0.05, (3) Hardy-Weinberg equilibrium P-value (PHWE) < 1e-6. After phasing and imputation, SNPs with (1) MAF < 0.01, (2) minor allele count < 20, (3) PHWE < 1e-6, (4) imputation quality (Rsq) < 0.7 were excluded. Before conducting GWAS, we removed the outliers whose metal concentrations were more than 4.5 inter-quartile ranges (IQRs) away from the median of each metal concentration.
Replication	For replication, we performed meta-analyses using GWAS summary statistics of the same metal from both FAMHES and MEWHC.
Randomization	This section is not covered in this study.
Blinding	This section is not covered in this study.

Reporting for specific materials, systems and methods

We require information from authors about some types of materials, experimental systems and methods used in many studies. Here, indicate whether each material, system or method listed is relevant to your study. If you are not sure if a list item applies to your research, read the appropriate section before selecting a response.

Materials & experimental systems

n/a	Involved in the study	
☒	☐	Antibodies
☒	☐	Eukaryotic cell lines
☒	☐	Palaeontology and archaeology
☒	☐	Animals and other organisms
☐	☒	Human research participants
☒	☐	Clinical data
☒	☐	Dual use research of concern

Methods

n/a	Involved in the study	
☒	☐	ChIP-seq
☒	☐	Flow cytometry
☒	☐	MRI-based neuroimaging

Human research participants

Policy information about studies involving human research participants

Population characteristics	In the end, we included 1,800 FAMHES participants and 688 MEWHC participants into our study. The median and inter-quartile ranges of the ages of the two groups are 36±16 and 44±9, respectively. 1,800 FAMHES participants are males and there were 468 males and 220 females in the 688 MEWHC participants. FAMHES participants were genotyped using the Illumina Omini one platform and MEWHC participants were genotyped using lnfinium Asian Screening Array-24 + v1.0 Kit (ASA).
Recruitment	Participants in FAMHES were recruited on the basis of a hospital physical examination population. Participants in MEWHC were recruited on the basis of Workers exposed to manganese. Although different genotype platforms were used to participants in FAMHES and MEWHC, we used the same approach in subsequent genotype imputation to reduce the differences between them. Considering the difference between FAMHES and MEWHC, we conducted the non-parametric test on the concentrations of each metal between the two cohorts via IBM SPSS Statistics 25. Finally, since there was no statistically significant difference in copper concentrations and manganese concentrations between the two cohorts, we conducted GWAS meta-analysis on the copper and manganese levels, respectively.

Ethics oversight

Our study was approved by the Ethics and Human Subject Committee of Guangxi Medical University. All the subjects provided written informed consent before participating in the study.

Note that full information on the approval of the study protocol must also be provided in the manuscript.
